# SifA SUMOylation governs *Salmonella* Typhimurium intracellular survival via modulation of lysosomal function

**DOI:** 10.1371/journal.ppat.1011686

**Published:** 2023-09-29

**Authors:** Hridya Chandrasekhar, Gayatree Mohapatra, Kirti Kajal, Mukesh Singh, Kshitiz Walia, Sarika Rana, Navneet Kaur, Sheetal Sharma, Amit Tuli, Prasenjit Das, Chittur V. Srikanth

**Affiliations:** 1 Regional Centre for Biotechnology, Faridabad, India; 2 Systems Immunology Department, Weizmann Institute of Science, Rehovot, Israel; 3 All India Institute of Medical Sciences (AIIMS), New Delhi, India; 4 Institute of Microbial Technology, Chandigarh, India; 5 Laboratory of Immunobiology, Universite´ Libre de Bruxelles, Gosselies, Belgium; 6 Department of Laboratory Medicine, Yale University, New Haven, Connecticut, United States of America; Purdue University, UNITED STATES

## Abstract

One of the mechanisms shaping the pathophysiology during the infection of enteric pathogen *Salmonella* Typhimurium is host PTM machinery utilization by the pathogen encoded effectors. *Salmonella* Typhimurium (*S*. Tm) during infection in host cells thrives in a vacuolated compartment, *Salmonella* containing vacuole (SCV), which sequentially acquires host endosomal and lysosomal markers. Long tubular structures, called as *Salmonella* induced filaments (SIFs), are further generated by *S*. Tm, which are known to be required for SCV’s nutrient acquisition, membrane maintenance and stability. A tightly coordinated interaction involving prominent effector SifA and various host adapters PLEKHM1, PLEKHM2 and Rab GTPases govern SCV integrity and SIF formation. Here, we report for the first time that the functional regulation of SifA is modulated by PTM SUMOylation at its 11^th^ lysine. *S*. Tm expressing SUMOylation deficient lysine 11 mutants of SifA (SifA^K11R^) is defective in intracellular proliferation due to compromised SIF formation and enhanced lysosomal acidification. Furthermore, murine competitive index experiments reveal defective *in vivo* proliferation and weakened virulence of SifA^K11R^ mutant. Concisely, our data reveal that SifA^K11R^ mutant nearly behaves like a SifA knockout strain which impacts Rab9-MPR mediated lysosomal acidification pathway, the outcome of which culminates in reduced bacterial load in *in vitro* and *in vivo* infection model systems. Our results bring forth a novel pathogen-host crosstalk mechanism where the SUMOylation of effector SifA regulated *S*. Tm intracellular survival.

## Author summary

Effectors are specialized proteins produced by bacteria that enable their entry, colonization, and survival within host. The effectors cross-talk with host molecular pathways for ensuring successful infection. Current study focuses on one such event during infection by gastroenteritis causing bacterial pathogen, *Salmonella* Typhimurium (*S*. Tm). During invasion, *S*. Tm develops a vacuole around itself with host membranes resulting in a protective structure called *Salmonella* Containing Vacuole (SCV). By effector function, particularly that of *Salmonella* induced filament protein A (SifA), it is known that SCVs evade lysosome mediated degradation in host cell. To issue these outcomes, SifA interacts and interferes with multiple host proteins. However, the details of these mechanisms are not fully understood. In eukaryotic cells, synthesized proteins undergo modifications collectively referred as ‘Post Translational Modifications’ (PTM), which diversifies their functionality. Here we tested if SifA utilizes one such host PTM machinery, i.e., SUMOylation for its function. Using cell culture and mice model we show PTM modification of SifA by SUMOylation. *Salmonella* encoding a SUMOylation defective SifA shows several weaknesses including a compromised SCV formation, enhanced lysosomal killing and reduced fitness. Thus, this work brings forth a novel bacterial mechanism, involving SUMOylation of effector SifA, required for *Salmonella* pathogenesis.

## Introduction

The Gram-negative bacterium *Salmonella* Typhimurium (hereafter to be referred as *S*. Tm) causes gastroenteritis in humans. Although, the infection clears out in a week’s span in healthy individuals, there is impending danger of bacteremia in immunocompromised individuals and infants. Infections caused by *S*. Tm are frequent, leading to a significant health burden in both developing and developed world [1]. The advent of multi drug resistant invasive strains of *S*. Tm also possesses additional threats notably in Sub-Saharan African countries and among children [2, 3]. Information regarding detailed molecular mechanisms are required to gain better insights behind the pathogenesis of this bacterium.

*S*. Tm pathogenicity is attributed to the presence of unique cluster of genes in its genomes making up the *Salmonella* pathogenicity islands. Among many SPIs, *Salmonella* pathogenicity island 1 (SPI1) and *Salmonella* pathogenicity island 2 (SPI2) are well explored [4]. A coordinated action of SPI-1 effector proteins aid in *S*. Tm entry into host along with induction of proinflammatory signaling mechanisms [5, 6].

Post uptake, *S*. Tm resides in a secluded membranous compartment called *Salmonella-* Containing Vacuole (SCV) which sequentially acquires markers of endocytic pathway. Early endosome antigen 1 (EEA1), GTPases Rab5 and Rab11 are initially recruited to SCV which are later interchanged with late endocytic markers Rab7, Lysosomal associated membrane proteins 1,2.3 (LAMPs) and vacuolar ATPases [7]. Sequestration of *S*. Tm within SCV is important since it enables intracellular replication and evasion of host defense mechanisms [8–10]. Members of the Rab family are crucial regulators of the intracellular membrane trafficking, thereby, they also modulate the fate of SCV [11]. Aided by effector functions, *S*. Tm are known to target the host GTPase Rab7 to modify SCV maturation [12]. Recently our group also showed that *S*. Tm manipulates Rab7 function by interfering with Rab7-SUMOylation, a post-translational modification mechanism [13]. Several crucial aspects of SCV biogenesis and its stability are majorly fine-tuned by an SPI-2 effector *Salmonella* induced filament A (SifA) protein [14]. SifA directs PLEKHM1 (Pleckstrin homology and RUN domain containing M1), Rab7 and HOPS (Homotypic fusion and protein sorting) complex to mobilize late endosomal membrane compartments for SCV generation [15]. PLEKHM1 directly interacts with both Rab7 and SifA, through its pleckstrin homology domain (PH domain) [16]. A ΔSifA mutant of *Salmonella* escapes SCV and fails to replicate in macrophages and thereby ends with compromised pathogenicity *in vivo* [17].

Tube like membranous extensions generated from SCV membrane, collectively called as *Salmonella*-induced filaments (SIFs), are generated during later stages of infection [18, 19]. SIFs are required for SCV nutrition and stability [20–22]. SifA function alters microtubule dynamics which is required for SIF formation [23] as well as recruitment of lysosome associated membrane protein 1 (LAMP1) on to the tubules [24, 25]. Furthermore, SifA forms a complex with SKIP (SifA and Kinesin Interacting Protein) also known as PLEKHM2 (Pleckstrin homology and RUN domain containing M2 protein) which enable regulation of plus-end- directed motor kinesin-1 on SIFs [26–28]. Though, SCV later fuses with lysosomes, they are devoid of recycling mannose-6-phosphate receptors (MPRs) responsible for trafficking lysosomal hydrolases into lysosomes [29, 30]. The GTPase Rab9 through its interaction with SKIP associates with MPRs for routing these hydrolases into lysosomes [31, 32]. During infection *S*. Tm interferes with Rab9-SKIP interaction and MPR pathway, together these mechanisms leads to blockade of lysosomal acidification [32, 33]. Thus, several mechanisms and functions crucial to Salmonellosis are attributed to SifA via its interaction with numerous host adapters. Being able to carry out several regulatory roles by SifA is a paradigm, the mechanistic details of which are still not fully understood. To accomplish multiple functions, effectors often utilize host PTMs.

Our group demonstrated that *S*. Tm control several aspects of host cell physiology by altering the SUMOylation machinery [34]. SUMOylation, a small ubiquitin like modifier system is known to post-translationally modify proteins and thereby affecting their function. SUMOylation being a powerful machinery can impact stability, folding, interaction and localization of their substrates [35]. SUMO protein exists as multiple isoforms in mammalian cells. SUMO-1 is 48% identical to SUMO-2 and shares 46% identity with SUMO-3, whereas SUMO-2/3 are 95% identical to each other. SUMO2/3 form poly chains on target proteins by conjugation, while SUMO-1 chains are only observed under *in vitro* condition [36, 37].

The global SUMOylome in hosts undergoes a major alteration during *S*. Tm infection, several of which belong to vesicular transport pathway [13]. Also, bacterial effectors from certain pathogens *Anaplasma* [38] and *E*. *chaffenis* [39] undergoing SUMOylation has been shown to be beneficial for pathogenesis. It is unclear if SifA or any of *S*. Tm effectors undergo PTM modification by SUMO. This study aims to identify whether SifA undergoes such processes and if so, how this modification could add complexity to *S*. Tm pathogenicity.

## Results

### Lysine 11 of SifA gets SUMOylated during infection

**Fig 1:**
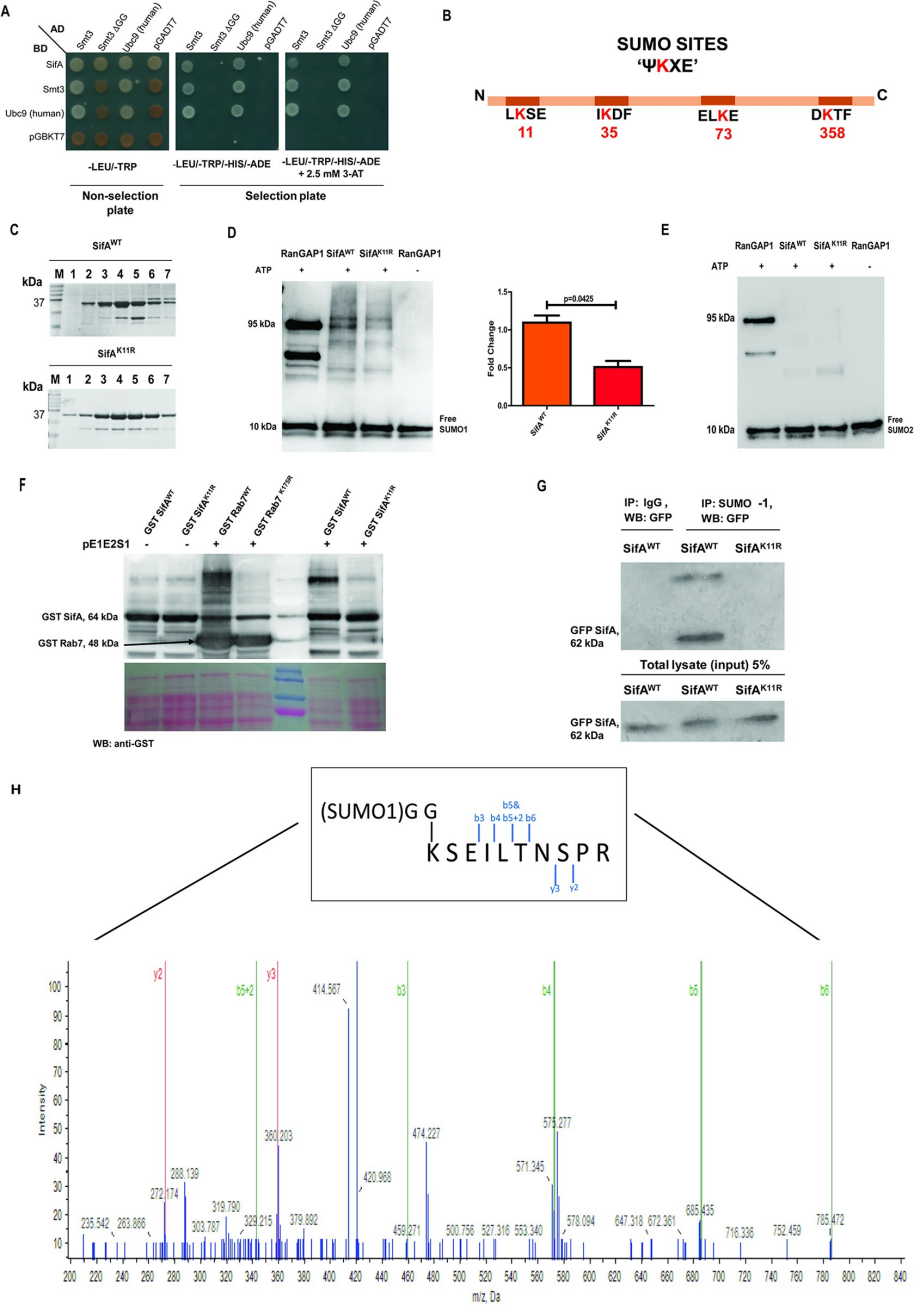
Lysine 11 of SifA gets SUMOylated during infection. **(A**) Yeast two hybrid **(**Y2H) assay indicating interaction between SifA and SUMO1, Smt3 (yeast SUMO1 ortholog), Smt3ΔGG (Smt3 with C-terminal di glycine deleted) and human Ubc9 (E2 enzyme of SUMO pathway). **(B)** Schematic representation of SifA primary sequence highlighting SUMO consensus motif and SIM sites. The lysine at 11^th^ position within a SUMO consensus site was predicted as a potential SUMOylation site with high probability by multiple in silico tools. **(C)** Fractions of purified SifA^WT^ and SifA^K11R^ corresponding to 37 kDa obtained after thrombin cleavage through chromatography by using superdox 200 column run on an SDS PAGE (12%) and Coomassie stained for expression analysis. Bands at 25 kDa correspond to GST carryover. **(D, E)** Resultant immunoblot of isolated proteins subjected to *in vitro* SUMOylation assay separately probed for both SUMO1 and SUMO2/3 isoforms. The results indicated the presence of higher molecular bands in wild type SifA incubated with SUMO-1 isoform (indicated in the densitometry plot, right panel), whereas the intensity of bands was remarkably less in K11R mutant. **(F)** In-bacto SUMO conjugation assay for GST tagged SifA expressed in *E*. *coli* BL21 along with pE1E2S1 plasmid encoding SUMO enzyme machinery. The blots were probed by anti-GST antibodies. Rab7 was used as a positive control [13] **(G)** Co-immunoprecipitation of eGFP-SifA expressed in HCT-8 cells pulled down by SUMO-1 antibody, succeeding immunoblot probed by anti-GFP antibody showed interaction only in wild-type SifA expressing lysates. **(H)** Spectra displaying fragmentation of SifA peptide bearing diglycine at K11. Inset represents schematic of SifA peptide conjugated to di-glycine of SUMO-1. The blots shown here are representative of at least three biological replicates.

Since, SifA and Rab7 reside in proximity in the SCV and Rab7 SUMOylation is prevented by *S*. Tm during infection [13], we investigated possible interaction of SifA with components of SUMOylation machinery. In our preliminary analysis, a yeast two hybrid assay was conducted to probe the interaction between SifA with Smt3, a yeast SUMO1 ortholog or Ubc9, the human E2 enzyme of SUMOylation pathway. The genes encoding SifA, Smt3, Smt3ΔGG and human Ubc9 were cloned in both yeast bait expression vector pGBKT7 expressing GAL4 DNA binding domain (BD) and pGADT7 vector designed to express GAL4 activation domain (AD) separately. Yeast cells (Y187, Clonetech’s matchmaker Y2H system) expressing both bait (pGBKT7) and prey vector (pGADT7) were confirmed by their growth on selection plates (Fig 1A). We observed that both Smt3 and Ubc9 showed interaction with SifA as seen by the growth of yeast on plates devoid of histidine and adenine. Notably, the mutant protein Smt3ΔGG, where C-terminal diglycine motif was deleted showed no growth. The diglycine motif at the c-terminal tail of SUMO paralogs participate in the iso-peptide bond formation with the lysine residue of the target protein, therefore the absence of this hinders SUMOylation [40, 41]. Screening was further carried on selection plates containing 2-Amino-1H-1,2,4-triazole (3-AT) at a concentration of 2.5mM adding stringency to the histidine-based growth selection. Similarly, selection comprising either one empty parent vector or between parent vectors led to complete inhibition of growth (Fig 1A). These data led us to conclude that SifA interacts with Smt3 and Ubc9. It was therefore important to examine if this interaction was a result of SifA undergoing SUMOylation.

The primary sequence of SifA was analyzed through various online ‘SUMO’ prediction software, including GPS-SUMO, JASSA and SUMO plot [42, 43], for identifying the presence of probable SUMO consensus sites. Multiple lysine harbored by SifA were picked by this software (Fig 1B). However, the search from all these above-mentioned programs collectively pointed at lysine11 (K11) lying within a SUMO consensus motif ΨKXE/D [44] displaying higher score in comparison to the cut-off (Table 1). Apart from the indicated SUMO consensus sites, primary sequence of SifA accommodate two probable SUMO interaction motif (SIM) sites spanning from amino acids 97–101 and 173–177 although this SIM site is not picked by the prediction software used in the study. SIM sites are short stretch of hydrophobic amino acids present in a protein through which it can interact with other proteins that are SUMOylated [45].

**Table 1:** Displaying potential SUMOylatable lysine(s) within SUMO consensus sites from primary sequence of SifA as predicted by indicated software. * Score above 0.75 are considered HIGH and where therefore selected.

Software	Lysine residue number (within SUMO consensus motif- Ψ-K-X-E)	Score	Cut-off	SUMO Interacting Motif site
GPS-SUMO	11	0.8678	0.72	No site detected
	73	0.8208	0.72	
SUMOplot	11	0.91	>0.75*	No site detected
	35	0.59	>0.75	
	358	0.15	>0.75	

To assess the possibility of SUMOylation at lysine 11, a mutant was generated with an arginine substitution (K11R) via site directed mutagenesis. The recombinant proteins wild type SifA (GST-SifA^WT^) and lysine mutant (GST-SifA^K11R^) were expressed in *E*. *coli* Rosetta strain and purified. The individual protein fractions obtained after thrombin cleavage from chromatographic column were run on SDS-PAGE where a band size corresponding to SifA (37 kDa) and GST (25 kDa) were visible (Fig 1C). *In vitro* SUMOylation assay was conducted with these purified fractions of SifA, using components from ENZO SUMOylation kit. Post reaction the mixture was run on SDS-PAGE and probed with anti-SUMO-1 and anti-SUMO2/3 antibody separately. As indicated in the immunoblot in Fig 1D, higher molecular weight bands were observed in SifA^WT^ when probed with SUMO1 antibody, whereas the intensity of bands was significantly less in SifA^K11R^, also quantified in the graph (Fig 1D). Presence of multiple bands in the blot may be due to SUMO-chain formation which is a common occurrence in *in vitro* SUMOylation reactions. Moreover, lack of any detectable band from the immunoblot probed by anti-SUMO2 suggests SUMO1-modification of SifA at lysine 11 (Fig 1E). Appearance of higher molecular weight bands in RanGAP1 (positive control) and clear lanes in reactions without ATP evinced the quality of SUMOylation reactions (Fig 1D and 1E). Purified RanGAP1 protein was used as positive control since it is known to undergo SUMOylation by both isoforms of SUMO. SUMOylation of RanGAP1 yields multiple bands observed above 40 kDa [46].

The above observations were further reinforced by another assay referred to as ‘in bacto-SUMOylation assay.’ This assay majorly relies on the expression of plasmid pE1E2S1 encoding SUMO machinery enzyme components (E1, E2) and SUMO1 (S1) besides the expression of target protein, SifA in *E*. *coli* [47]. The pE1E2S1 plasmid along with constructs encoding SifA^WT^ or SifA^K11R^ were co-transformed in *E*. *coli* BL21 cells. The lysates from these cells were run on SDS-PAGE and immunoblotted. As depicted in Fig 1F, a prominent band around 120 kDa was observed, indicating possible SUMO-modification of SifA, only in sample comprising co-transformed wild type SifA and pEIE2S1 construct, but not with those with pE1E2S1 and K11R mutant. As seen from our previous assays, presence of multiple bands in the blot may be due to SUMO-chain formation. The mammalian protein Rab7 and its corresponding SUMO mutant, Rab^K175R^ were also included here as a positive control for the assay respectively [13] (Fig 1F). As anticipated, higher mobility bands in case of Rab7 but not for Rab^K175R^ was observed.

In order to validate these observations *in vivo* in mammalian cells, a co-immunoprecipitation (co-Ip) assay was conducted using human intestinal epithelial cells, HCT-8. HCT-8 cells were transfected by plasmids bearing both SifA^WT^ and SifA^K11R^ genes cloned in mammalian peGFP-C1 vector separately. After 24 hours of transfection, the transfected cells were subjected to infection by ΔSifA mutant for 7 hours. Immunoprecipitation was carried by anti-SUMO1 antibody followed by immunoblotting with anti-GFP antibody. A band corresponding to GFP-SifA^WT^ around 62 kDa and prominent upper band, representing SUMOylated version of SifA, was observed only in wild type samples. Notably, in line with our above results, we did not observe any bands in SifA^K11R^ transfected samples making it indistinguishable from isotype control (Fig 1G). The expression of SifA^K11R^ protein was observable in the input sample (Fig 1G). To investigate whether SifA undergoes SUMOylation during infection, HCT-8 cells were infected by SifA^WT^ and SifA^K11R^ complemented strains in ΔSifA (pSifA^WT^ & pSifA^K11R^ respectively) using prokaryotic expression vector pACYC-184. The infection was allowed to proceed till 7 hours at a MOI (Multiplicity of Infection) of 1:40. A faint band corresponding to SUMOylated SifA was identified only in SifA^WT^ infected sample as shown in S1A Fig. Further the validity of SifA SUMOylation at site 11 was validated by mass-spectrometry (MS peptide summary provided in S1 Data file), where the reaction consisting *in vitro* SUMOylation of SifA was subjected to mass-spectrometry. The spectra corresponding to the peptide bearing SUMOylated SifA at lysine 11 is shown in Fig 1H, also the fragmentation for same peptide is shown in inset.

Finally, an *in-situ* detection of SUMO modified SifA in cells were accomplished through a proximity ligation assay (PLA). This assay was conducted in HeLa cells transfected by peGFP-C1 plasmids containing SifA^WT^ or the non-SUMOylatable K11R mutant SifA (hereafter to be also referred as SUMOylation deficient SifA) for 24 hours. The transfected cells were probed by anti-GFP antibody (rabbit) and anti-SUMO-1 antibody (mouse). PLA mainly relies on the attachments of oligonucleotide labeled secondary antibodies against the primary that will only be joined and ligated if the interacting proteins are in proximity. The interaction is expected to result in the formation of a closed circular DNA template which is eventually amplified and detected by detection oligos which can be visualized as discrete puncta (S1B Fig). In the control sample, one primary antibody (anti-GFP) was omitted. As shown in the S1C Fig, the puncta from individual cells quantified suggested interaction between SifA and SUMO-1. The number of punctae was observed highest in SifA^WT^ transfected cells in comparison to those with SifA^K11R^ (S1C Fig), while, there was no signal in the negative control (left panel). Based on the results from all these above experiments, we concluded that SifA undergoes SUMO-modification by SUMO-1 isoform at lysine 11.

### Ectopically expressed SifA^K11R^ displays compromised stability

As it is well established that SUMOylation can potentially impact substrate’s stability, folding, interaction or/and compartmentalization [35], we therefore set out to investigate the biological consequences of SifA SUMOylation.

**Fig 2:**
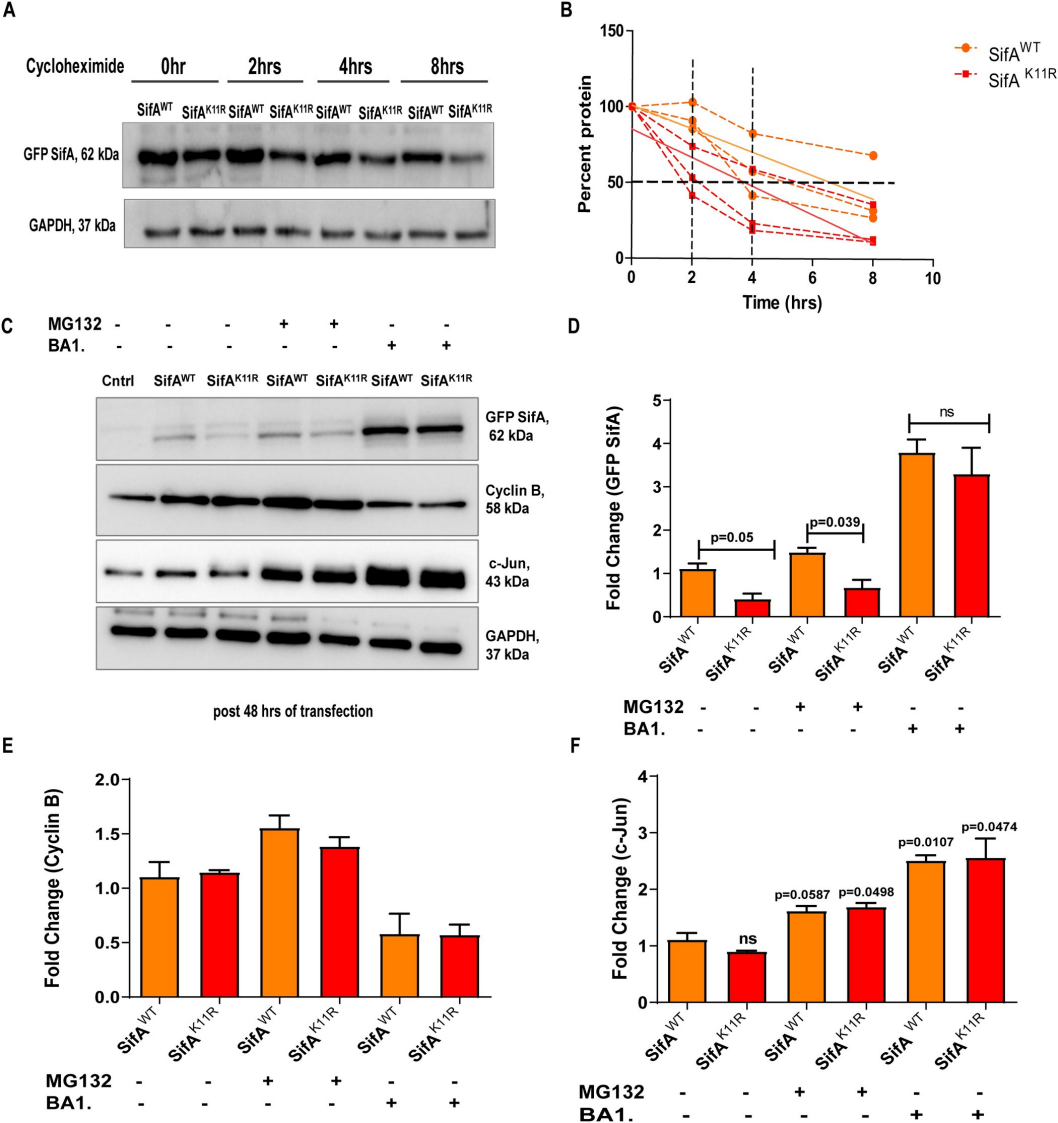
Ectopically expressed SifA^K11R^ protein is unstable in comparison to wild-type. **(A)** Cycloheximide (100μg/ml) was incorporated into SifA^WT^ and SifA^K11R^ transfected HCT-8 cells after 24 hours of transfection. Expression levels of both proteins are indicated till 8 hours of treatment duration (cyclo chase). **(B)** Decay curve indicating the stability of both proteins. The level of each protein at time 0 was set as 100% and the percentage of protein remaining at each time point from three independent experiments was calculated and plotted. **(C)** Blots representing the expression levels of wild-type and SUMO mutant SifA along with control proteins cyclin B and c-Jun and its **(D)** relative quantification on proteasomal inhibitor MG132 (20μM) treatment and on lysosomal acidification inhibitor bafilomycin A1 (100nM) treatment. Normalized expression levels of **(E)** cyclin B and **(F)** c-Jun in response to drug treatments. All statistics had been performed by students unpaired t-test. The blots shown here are representative of at least three biological replicates.

HCT-8 cells transfected by peGFP-C1 plasmids containing SifA^WT^ or SifA^K11R^ were monitored for indicated time periods as shown in supplementary S2A Fig. There were no notable differences in expression at 24- or 36-hours post transfection between SifA^WT^ and SifA^K11R^ (S2B Fig). However, at 48 hours post transfection, compared to SifA^WT^ a significant decrease in pools of SifA^K11R^ was observed as shown in S2B Fig. Next, cycloheximide chase assay was executed to determine the steady state stability and half-life of both proteins [48]. To accomplish this, cycloheximide at a concentration of 100 μg/ml was incorporated into transfected HCT-8 cells succeeding 24 hours of transfection (Fig 2A). The decay curve represents amount of remaining percent protein acquired from three independent chase (individual replicates are plotted) at each time point in comparison to start of the experiment (time 0). As it is evident from the curve (Fig 2B), the stability of SifA^K11R^ was considerably less compared to the wild type protein beginning at 2 hours. At 4 hours the pools of SifA^K11R^ were at 25% of the initial levels (two replicates), whereas the levels of SifA^WT^ were around 50% of initial (two replicates). Now to ascertain the pathway leading to the degradation of SifA^K11R^, transfected cells were treated with MG132 (inhibitor of ubiquitin proteasomal pathway) or bafilomycin A1 (blocker of lysosomal degradation). It was evident that the levels of both the proteins did not alter significantly upon MG132 treatment (Fig 2C and 2D), only a mild increase in both proteins were seen. However, treatment of bafilomycin A1 (BA1.) restored the levels of SifA^K11R^ in addition to significant increase in the levels of both proteins compared to untreated samples (Fig 2C and 2D). Thus, the relative pools of wild type and SifA^K11R^ were identical in presence of this drug indicating a role of lysosomal activity in regulation SifA^K11R^ expression. Additional proteins cyclin B and c-Jun were included as controls. Cyclin B is known to undergo proteasomal lysis [49], thus showing maximum recovery in MG132 treated samples (Fig 2E), and similarly c-Jun is known to undergo degradation via both the pathways [50] (Fig 2F). Next, live cell imaging was carried out in SifA^WT^ or SifA^K11R^ transfected HCT-8 cells, to assess the co-localization between SifA and lysosomes. Lysosomes were stained by the cell permeable fluorescent dye lysotracker red (S2C Fig). Live cell snaps acquired via confocal imaging displayed diminished GFP fluorescence (green channel) in case of GFP SifA^K11R^ (S2C and S2D Fig) accounting to their diminished stability. Interestingly, we have also seen increased lysotracker intensity in case of GFP SifA^K11R^ in comparison to GFP SifA^WT^ (S2E Fig), although both proteins co-localized with lysosomes in similar fashion (S2F Fig). Over expression of SifA results in increased lysosomal activity compared to vector control, while lysotracker staining intensity further escalated in presence of GFP SifA^K11R^ hinting the role of SifA in modulating lysosomal function through its SUMOylated lysine 11. Hence, the data from above experiments implied that stability of ectopically expressed SifA^K11R^ is lesser due to active lysosomal activity compared to its wild type counterpart.

### Infection with *Salmonella* bearing SUMO deficient SifA results in altered expression of host proteins, SKIP and MPR

**Fig 3:**
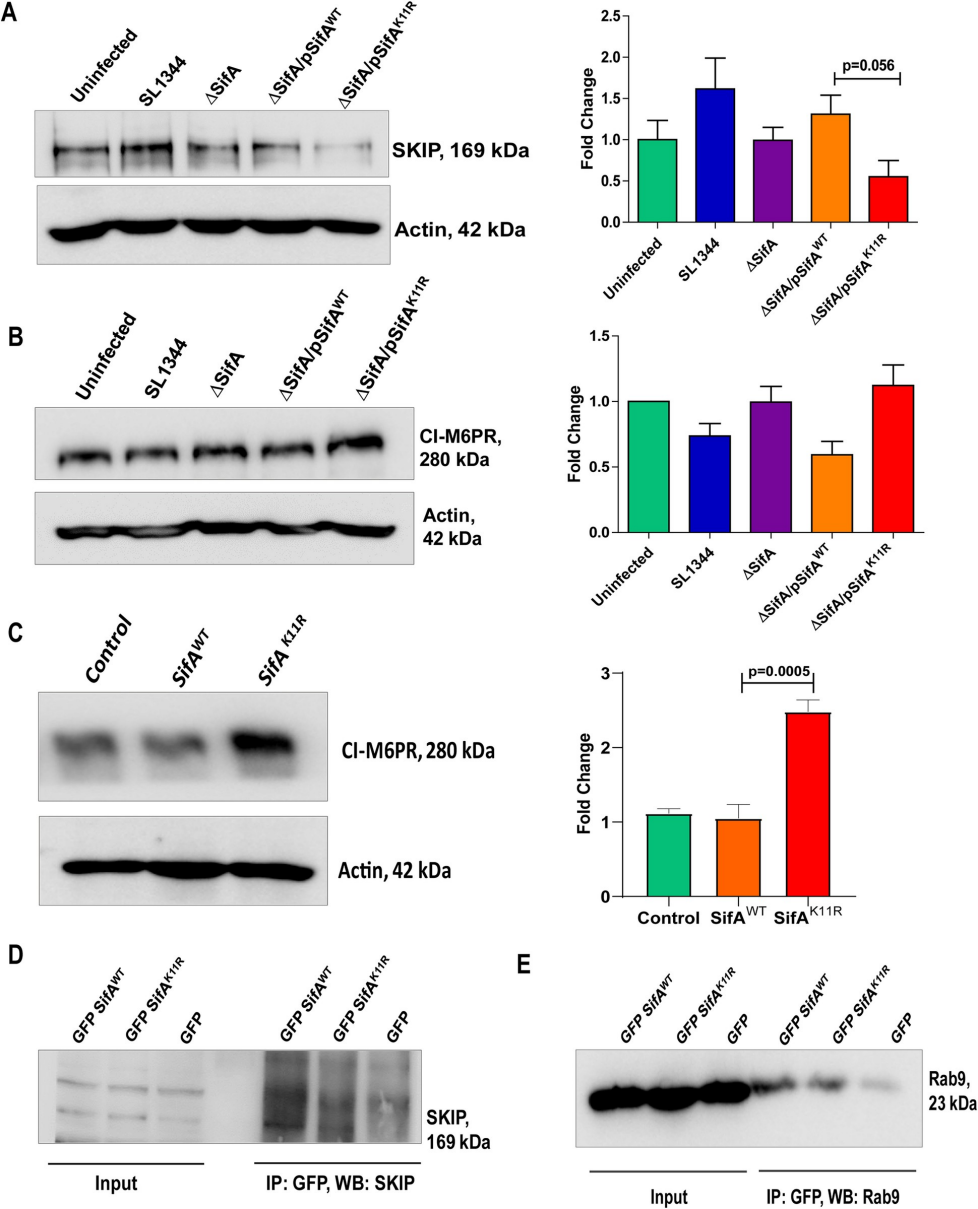
SifA-K11R infection leads to alteration in expression levels of host proteins involved in lysosomal acidification. **(A)** PLEKHM2 (SKIP) expression levels and their quantification in response to infection with depicted strains of *Salmonella*. **(B)** CI- M6PR expression levels in response to infection by indicated strains of *S*. Tm. **(C)** Immunoblot and its densitometric analysis representing CI-M6PR expression in transfected samples. Control refers to lysates prepared from non-transfected samples. **(D)** Co-IP assays showing interaction of SifA^WT^ and SifA ^K11R^ with SKIP protein and **(E)** small GTPase Rab9 from transfected HCT-8 cells. The blots shown here are representative of at least three biological replicates.

Taking into consideration, the role of lysosomes in regulating stability of SifA from our previous findings and the fact that one of major functions of this effector is protection of the bacterium from lysosomal degradation, we decided to decode how each downstream adapter involved in this process respond to SifA SUMOylation. As mentioned in the introduction, SifA is involved in subverting retrograde trafficking of mannose-6-phosphate receptors (MPRs) hence inhibiting lysosomal acidification. It is known that SifA-SKIP complex is formed during *Salmonella* infection enables sequestration of Rab9 protein leading to misrouting of MPRs [32]. Also, the host interactor of SifA, PLEKHM2 (SKIP) is regarded as a negative regulator of lysosomal activity [32] and recently its expression was known to be modulated by certain strain of *Mycobacteria* [51]. We began by examining whether SKIP expression gets modulated during *Salmonella* infection. Accordingly, HCT-8 cells were infected by the following strains of *Salmonella*; SL1344 (wild type), ΔSifA (SifA gene knock-out in SL1344 background), ΔSifA complemented with SifA^WT^ (referred as SifA^WT^) or SifA^K11R^ (referred as SifA^K11R^) (cloned in pBH-HA vector) individually at a MOI of 1:40 for 4 hours. Samples infected by SL1344 showed some increase (although not statistically high) in SKIP levels compared to lysates generated from uninfected control, but the levels did not rise in ΔSifA infected samples (Fig 3A). Notably, there was a decrease in SKIP expression in SifA^K11R^ infected samples (p value = 0.056) (Fig 3A). Similarly, the same samples were probed for the presence of Cation Independent mannose 6-phosphate receptor (CI-M6PR). The blot in Fig 3B showed the levels of CI-M6PR are high in ΔSifA and SifA^K11R^ infected samples in comparison to their respective wild-type proteins. Lysates obtained from ectopically expressed SifA^WT^ and SifA^K11R^ in HCT-8 cells after 24 hours of transfection exhibited a drastic variation in the expression of protein CI-M6PR (Fig 3C). The levels of CI-M6PR protein were significantly enhanced in case of SifA^K11R^ expressing lysates (Fig 3C). We surmised that, unlike its wild type counterpart, SifA^K11R^ is deficient in the known function of preventing lysosomal acidification. Further, we also checked the ability of SifA^K11R^ to interact with its adapters SKIP and Rab9 via independent co-IP experiments from transfected HCT-8 cells. As depicted in Fig 3D, band of SKIP protein from IP fraction corresponding to SifA^K11R^ pull down was fainter compared to SifA^WT^. Likewise, the interaction between SifA^K11R^ and Rab9 necessary for trafficking of lysosomal hydrolases was also lesser compared to wild type (Fig 3E), hinting inability of SUMO mutant of SifA in sequestering Rab9 through its interaction with SKIP. Progressing, Rab9 CI-M6PR co-localization was compared during an infection with complemented strains SifA^WT^ and SifA^K11R^. Co-localization assays were conducted in HeLa cells, as this cell line is widely employed for imaging studies related to *Salmonella* infection [52–54]. HeLa cells were infected by SifA^WT^ or SifA^K11R^ separately at a MOI of 1:40 for a duration of 7 hours, after which these infected cells were fixed, stained for CI-M6PR (green channel), Rab9 (red channel) cell nuclei and indicated strains were stained by DAPI (blue channel) (S3A Fig). The combined analysis from individual cells portrayed a statistically significant increase in co-localization between CI-M6PR and Rab9 in case of infected cells by SifA^K11R^ compared to wild-type. Since the co-localization between Rab9 and CI-M6PR were affected, the distribution of both these proteins over SCVs were also assessed. CI-M6PR receptor was found to be better localized to SCV marker LAMP1 on SCVs from SifAK^11R^ infected cells (5.5 hours pi) (S3B Fig). The distribution of Rab9 on SCVs from all four strains (S4A and S4B Fig) showed no change. Thus, the expression and colocalization of CI-M6PR were differently affected during SifA^WT^ and SifA^K11R^ infection. The inference from above observations concur SUMOylation of SifA at 11^th^ lysine promotes misrouting of Rab9-MPRs trafficking. Thus, it is hypothesized that SifA^K11R^ is less potent of providing protection to SCV from digestion by lysosomal hydrolases. The predicted outcome in the current scenario stipulates escalated lysosomal activity in SifA^K11R^ infected cells compared to SifA^WT^.

### SUMOylation of SifA is required for preventing lysosomal acidification and eventual lysosomal clearance of *Salmonella*

**Fig 4:**
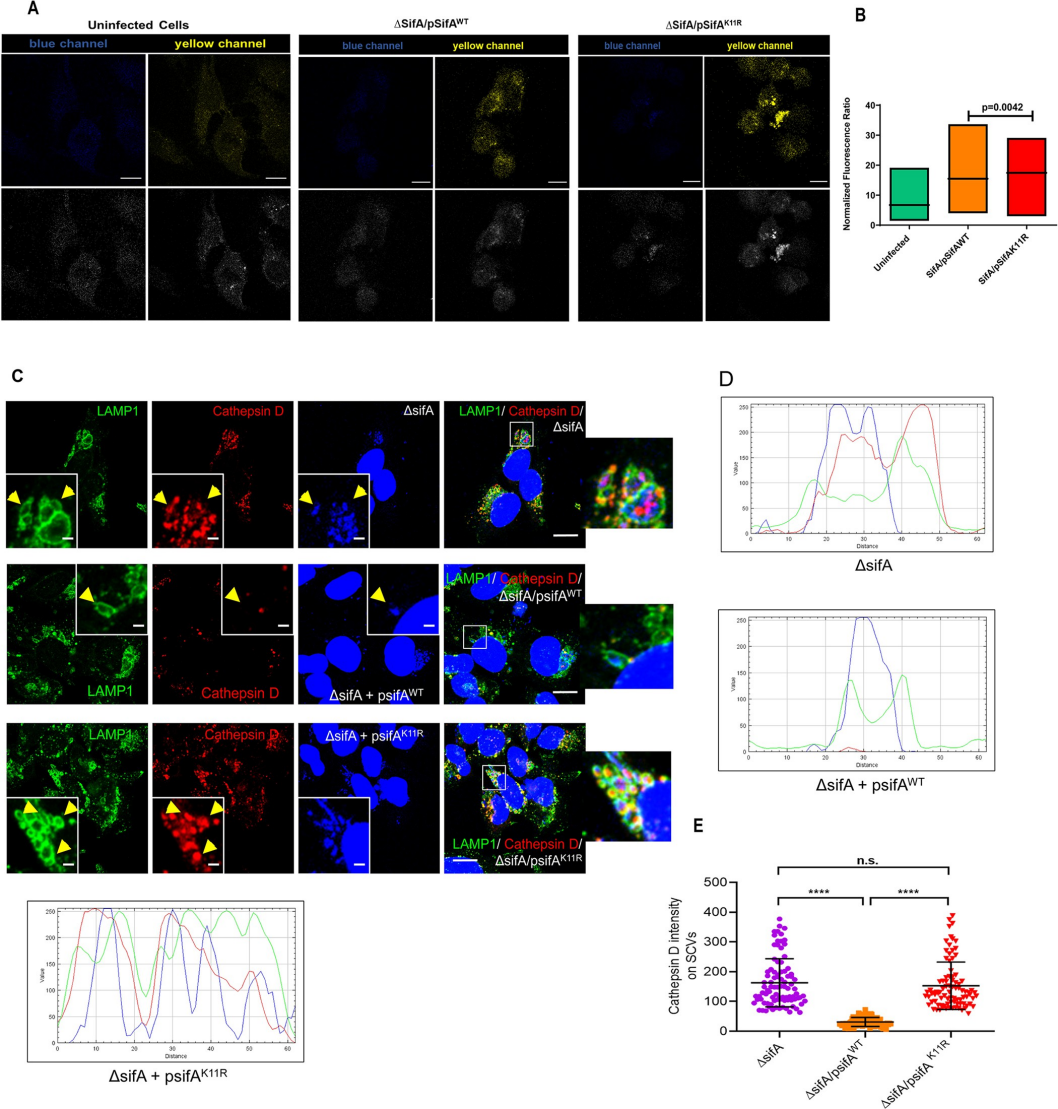
SUMOylation of SifA is required for preventing lysosomal acidification and eventual lysosomal clearance of *Salmonella*. **(A)** Images of HeLa cell fields infected by shown strains acquired for lysosomal activity measurement using lysosensor yellow/blue which emits bright yellow fluorescence in response to acidified environments (scale 25 micron), the images are shown in both colored (top panel) and grey scale (bottom panel). **(B)** Quantification and comparison of yellow fluorescence (acidic environment) emitted from lysosensor stained cells across indicated samples. **(C)** Images of cathepsin D localization over SCVs obtained after infection by indicated strains in HeLa cells for 4 hours. **(D, E)** Quantification of cathepsin D (red) signal overlaps with LAMP1 (green) and DAPI and its co-localization over SCVs.

HeLa cells were treated with a ratiometric probe called lysosensor yellow/blue DND to monitor the difference in lysosomal acidity across the samples. The dye fluorescence shifts from blue to bright yellow as the pH shifts from neutral to acidic environment [55, 56]. As shown, in Fig 4A, from the fields corresponding to infection with SifA^WT^ and SifA^K11R^, the intensity of yellow panel was higher in latter compared to the former, thus implying that SifA^K11R^ is incapable of lowering the lysosomal hydrolase activity. Fluorescence from uninfected cells was also shown for reference (Fig 4A and 4B). Further, we quantitated the amount of a lysosomal hydrolase cathepsin D over SCV. HeLa cells were infected by ΔSifA, SifA^WT^ or SifA^K11R^ at the regular MOI and SCVs from infected cells were co-stained for cathepsin D and LAMP1 (Fig 4C). The presence of cathepsin D (red) was indicated graphically as signals overlapping on SCV marker LAMP1 (green) and bacteria (blue channel) on selected SCVs as ROIs (Fig 4C and 4D). From these images it was evident that cathepsin D co-localized more on SCVs from ΔSifA and SifA^K11R^ infected cells, whereas as they were barely present on SCVs from SifA^WT^ infected cells (Fig 4E). From here on, we concluded that SUMOylation of SifA is necessary for *S*. Tm to overcome degradation by lysosomal hydrolases.

### Host cells infected by *S*. Tm with SUMO deficient SifA display fewer SIFs

**Fig 5:**
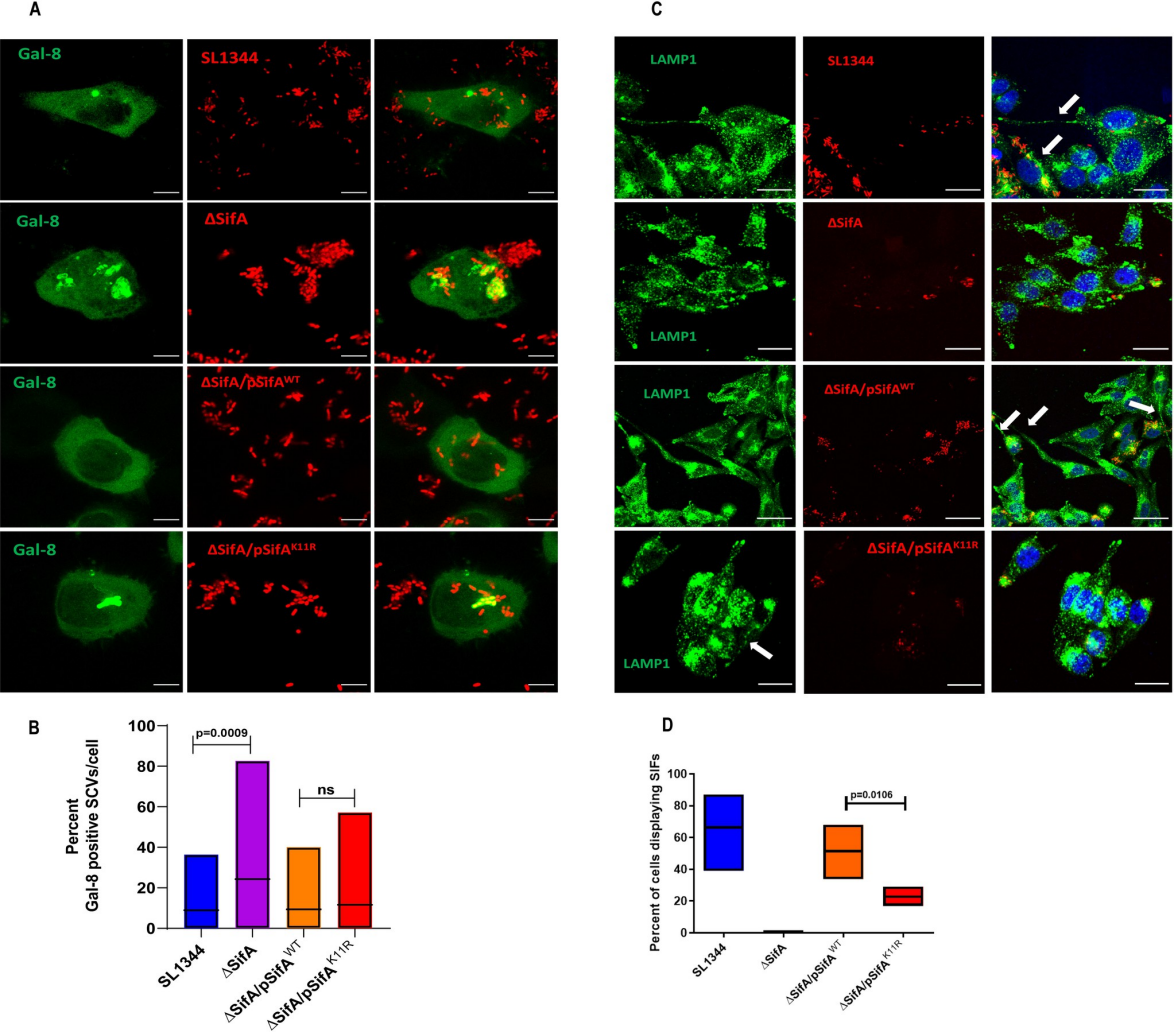
Cells infected by SifA^K11R^ displays fewer SIFs. **(A)** Images from HeLa cells infected by indicated strains transformed by mCherry plasmid compared for their ability to form SCVs 1hour post infection. The integrity of SCVs was assessed by detecting the presence of the marker galectin-8 via live cell imaging (scale 8 micron). **(B)** Gal-8 positive rods were enumerated out of total rods in a cell and expressed as percent. **(C)** Image fields with cells infected with indicated strains stained by SIFs marker LAMP1 probed after 10 hours post infection (scale 50 micron). **(D)** Quantitative representation of percent of cells containing SIFs from samples infected by varied strains of *Salmonella*.

As mentioned earlier, *S*. Tm pathogenicity relies greatly on the ability to generate SCV and SIFs in the endocytosed host cells, both of which are majorly attributed to effector SifA [14, 23]. These phenotypes were surveyed in cells infected with SifA^WT^ and SifA^K11R^ to understand the role of SUMOylation in SifA function. The protein galectin-8 is regarded as an indicator of SCV integrity. Galectin-8 binds to exposed host glycans on damaged SCVs activating xenophagy in host [10]. Galectin-8 (Gal-8) GFP plasmid transfected HeLa cells were infected by complemented strains possessing SifA^WT^ or SifA^K11R^ or SL1344 or ΔSifA at an MOI of 1:40. Live cells were imaged 1 hour pi to determine the acquisition of galectin-8 protein on SCV. From Fig 5A it was evident that gal-8 (green channel) localized on SCV (red channel) distinctly during infection by shown strains which is also represented graphically in Fig 5B. ΔSifA had high amount of galectin-8 over SCVs compared to wild type, indicating deformed vacuole formation, followed by SifA^K11R^, although there was no drastic difference in their accumulation compared to SifA^WT^ (Fig 5B). Proceeding further, infected HeLa cells were enumerated for visible SIFs to understand the effect of SifA SUMOylation on SIF generation. In general SIFs are formed at later hours of infection by fusion of SCV with late endocytic compartments carrying membranes enriched in glycoprotein LAMP1 [57]. Thus, to enable visualization of SIFs, HeLa cells after 10 hours pi, were fixed and stained for the glycoprotein marker LAMP1. Image fields displaying LAMP1 (green channel) captured from more than forty fields infected by indicated strains are shown in Fig 5C. The white arrows specify elongated SIFs which appear like thin strands of thread. The number of SIFs counted from acquired frames corresponding to each strain were calculated in percentage and are presented graphically in Fig 5D. Overall, from the data it was clear that presence of SIFs was maximally seen in cells infected by bacteria expressing SifA^WT^. Besides no SIFs were produced in cells in response to ΔSifA infection. Intriguingly, cells infected by bacteria expressing SifA^K11R^, akin to ΔSifA, showed substantially fewer SIFs compared to the wild type strains. In view of the above data, we concluded that SUMOylation of SifA functionally regulates critical aspects related to *S*. Tm pathogenesis.

### SifA SUMOylation is necessary for *Salmonella* replication and virulence

**Fig 6:**
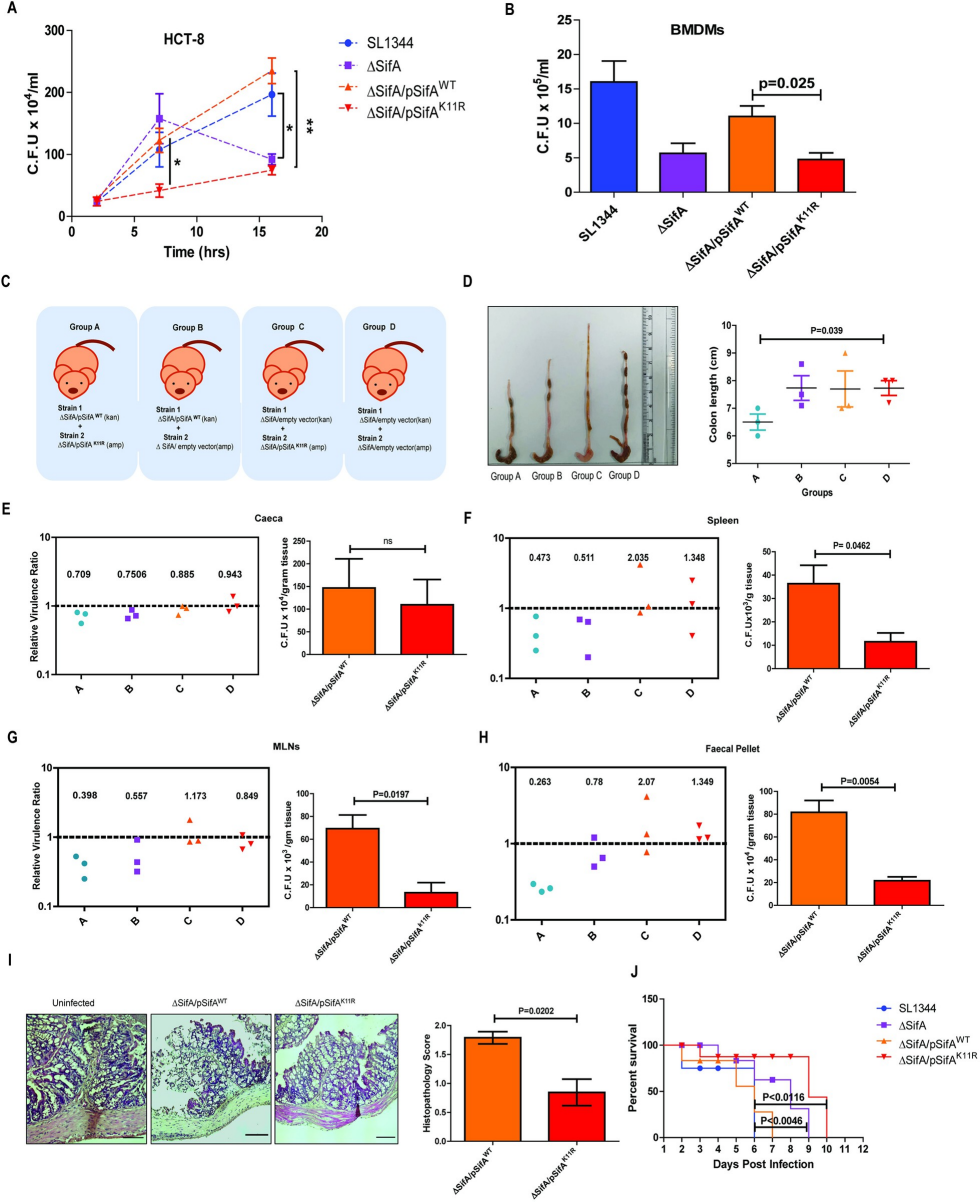
SifA SUMOylation is necessary for implementing virulence. **(A)** Gentamycin protection assay results indicating bacterial burden in HCT-8 cells infected by the shown strains at 2,7- and 16-hours post infection (* denotes p value of 0.0215 and ** equals p value of 0.0018). **(B)** Intracellular bacterial load from isolated primary BMDMs infected by shown strains at 16 hours post infection. **(C)** The schematic describing the mice groups infected with the indicated strains. CI assay involves infecting the animal with mixed inoculum consisting strain 1 (WT) and strain 2 (K11R) in equal ratio. **(D)** The colon morphology comparison from infected animals across the groups. Graphical representation of colon length from shown groups with significant variation in group A (pSifA^WT^ + pSifA^K11R^) and D (Vector control). **(E-H)** Relative virulence ratio calculated by the equation described in the text. CI with mean index is displayed for bacterial counts obtained from caeca, spleen, MLNs, and fecal pellet. The bar graph at the right compares the bacterial load plotted from mice individually infected by the indicated strains. **(I)** H and E studies showing increased intensity of inflammation in pSifA^WT^ infected mice proximal colon sections. The histopathology of the colon was quantified by considering parameters like epithelial damage, goblet cell loss and immune cell infiltration which were scored blindly by a trained pathologist. **(J)** The survival curve representing no. of days lived by every infected mouse from all groups. Each drop signifies death of an animal in the group. Mice infected by ΔSifA and pSifA^K11R^ strains showed prolonged survival owing to attenuated virulence.

Virulence of bacterial pathogens is mainly dependent on their ability to replicate and disseminate inside their host. We therefore set forth to investigate the survival and virulence potential of wild type SifA and SUMO mutant SifA expressing *Salmonella* strains. Both the wildtype SifA complemented ΔSifA strain (SifA^WT^), and SUMOylation deficient SifA complemented ΔSifA strain (SifA^K11R^) showed similar SifA expression (S5A Fig). Furthermore, SL1344, ΔSifA, SifA^WT^ and SifAK^11R^ followed similar growth kinetics (S5B Fig). Gentamycin protection assay (GPA) was performed in HCT-8 cells infected with above mentioned four strains to investigate intracellular bacterial load. Cells at 2 hours post-infection (pi) revealed no change in colony forming units (CFU) (Fig 6A). However, at 7 hours pi, CFU from SifA^K11R^ infected cells was considerably lesser compared to other strains (Fig 6A). The replication of ΔSifA at this point was very high, as expected due to hyper replication of this strain owing to loss of vacuole [58]. After 16 hours pi, the difference in intracellular bacterial load from cells infected by both ΔSifA and SifA^K11R^ was significantly lower in comparison to their respective wild type controls (Fig 6A). Aside from epithelial cells, we also carried out GPA in isolated primary bone marrow derived macrophages (BMDMs) from mice. *ex vivo* infection of BMDMs by the above-mentioned strains for duration of 16 hours, yielded similar pattern where strain SifA^K11R^ replicated at a slower rate in comparison to SifA^WT^ (Fig 6B). Finally, the virulence potential of SifA^WT^ and SifA^K11R^ were further tested in other cell lines HeLa (S5C Fig), CaCo2 (S5D Fig) and RAW 264.7 (S5E Fig) post 16 hours pi. The pattern observed was analogous to the data from HCT-8 and macrophages. Thus, it was evident that SifA^K11R^ exhibited a severely compromised replication *in vitro* in a range of different cell types. Since most of our experiments focused on K11 mutant, we were intrigued by the fact whether the same lysine is targeted by ubiquitin machinery. Multiple bands that can be seen in the blot across all the lanes may be representing the poly-ubiquitination in other sites (S5F Fig). Notably, a unique and prominent band was seen exclusively in GFP-SifA^WT^ suggesting lysine 11 is also targeted by ubiquitin machinery (S5F Fig). Now to answer the obvious question, whether the phenotypes associated with SifA^K11R^ mutant is due to SUMOylation or ubiquitination, we performed a bacterial burden assay after knocking down Ubc-9, the sole E-2 enzyme of SUMOylation pathway (S5G Fig). When Ubc-9 was knocked down, SifA^WT^ replicated at similar rates compared to pSifA^K11R^ suggestive of the fact that SUMOylation is required for SifA function (S5H Fig).

The attenuated replication of *S*. Tm bearing SifA^K11R^ detected *in vitro* prompted us to examine the behavior of this strain in mice models of *Salmonella* infection. Streptomycin pre-treated C57BL/6 female mice model was adapted to carry out all the infections. A competitive index (CI) assay was conducted [59] to assess the fitness of SUMOylation deficient SifA^K11R^ strain in comparison to SifA^WT^. To actualize this assay, ΔSifA complemented with SifA^WT^ cloned in pET28a vector (vector 1, kanamycin selection) was mixed with SifA^K11R^ complemented in ΔSifA via pET21a vector (vector 2, ampicillin selection) in equal ratio to achieve a MOI of 5x10^7^ CFU/ml. The parental *Salmonella* strain SL1344 used here is streptomycin resistant. The input inoculum contained equal volume of both cultures since they multiplied at same rate in LB media. Streptomycin treated mice post 24 hours, were fed with the above stated mixed cultures by oral gavage, for a total duration of 48 hours after which animals were euthanized and tissue lysates plated for CFU enumeration. The panel (Fig 6C) indicates the categorized mice groups (N = 3) fed with the corresponding strains as shown. Colon harvested from these mice groups were assessed for their morphology, which exhibited maximum inflammatory features in group A (bearing pSifA^WT^ + pSifA^K11R^) whereas these features were minimally seen in group D (bearing Vector1+Vector2) (Fig 6D). Furthermore, the tissue homogenate from caeca, spleen, mesentric lymph nodes (MLNs), and fecal pellets were used to calculate CFUs on McConkey agar with appropriate selection markers (kanamycin for SifA^WT^ and ampicilin for SifA^K11R^). The following formula was used to calculate the Competitive Index:

$$\mathrm{CI}=\frac{\mathrm{CFU}\;\mathrm{mutant}\;(\mathrm{output})}{\mathrm{CFU}\;\mathrm{wt}\;(\mathrm{output})}|\frac{\mathrm{CFU}\;\mathrm{mutant}(\mathrm{input})}{\mathrm{CFU}\;\mathrm{wt}\;(\mathrm{input})}$$

The CI signifying relative virulence ratio calculated as mentioned above from organs are shown in Fig 6E–6H. An index value close to 1 suggests both the strains replicate at equal rate, while a value farther from 1 indicate dominance of one strain over other in fitness. The mean index of CI values from three mice is also shown in the graph. As evident from these graphs, both SifA^WT^ and SifA^K11R^ strains were able to colonize and multiply similarly in mice gut like other groups. However, the index values for group A varied from group D significantly in other organs checked. Furthermore, the index values were comparable between groups A and B. However, the values for groups C and D were distinct from groups A and B meaning wild type strain outcompeted K11R which replicated and disseminated identical to ΔSifA strain. To support the above data, CFU involving single infections (5x10^7^ CFU of individual strain per mice) performed in same organs under similar conditions, were also added beside their corresponding CI plots (Fig 6E–6H). Differences in bacterial load were largely observed in MLNs and faecal pellet. Histopathological analysis involving Hematoxylin and Eosin (H & E) studies done in proximal colon tissue sections from mice infected by indicated strains showed severity of inflammation varied between wild type and SUMO deficient SifA (Fig 6I). The histopathology score tested was higher for SifA^WT^ compared to SifA^K11R^. Finally, a survival assay was administered comprising SL1344 and ΔSifA in addition to complemented strains for evaluating their virulence in mice. Mice groups orally infected by depicted four strains (N = 4) separately at the same time were left monitored to decide the progression of infection leading to bacteremia and death in these animals. The curve in Fig 6J indicated the mice infected with complemented strain SifA^K11R^ survived for more days (till day 10), like ΔSifA strain. However, mice groups infected by the wild type strains succumbed to infection much earlier. These data sets identified that mutant K11R of SifA bearing strain is comparably attenuated in virulence and potential to spread Salmonellosis in animals.

**Fig 7:**
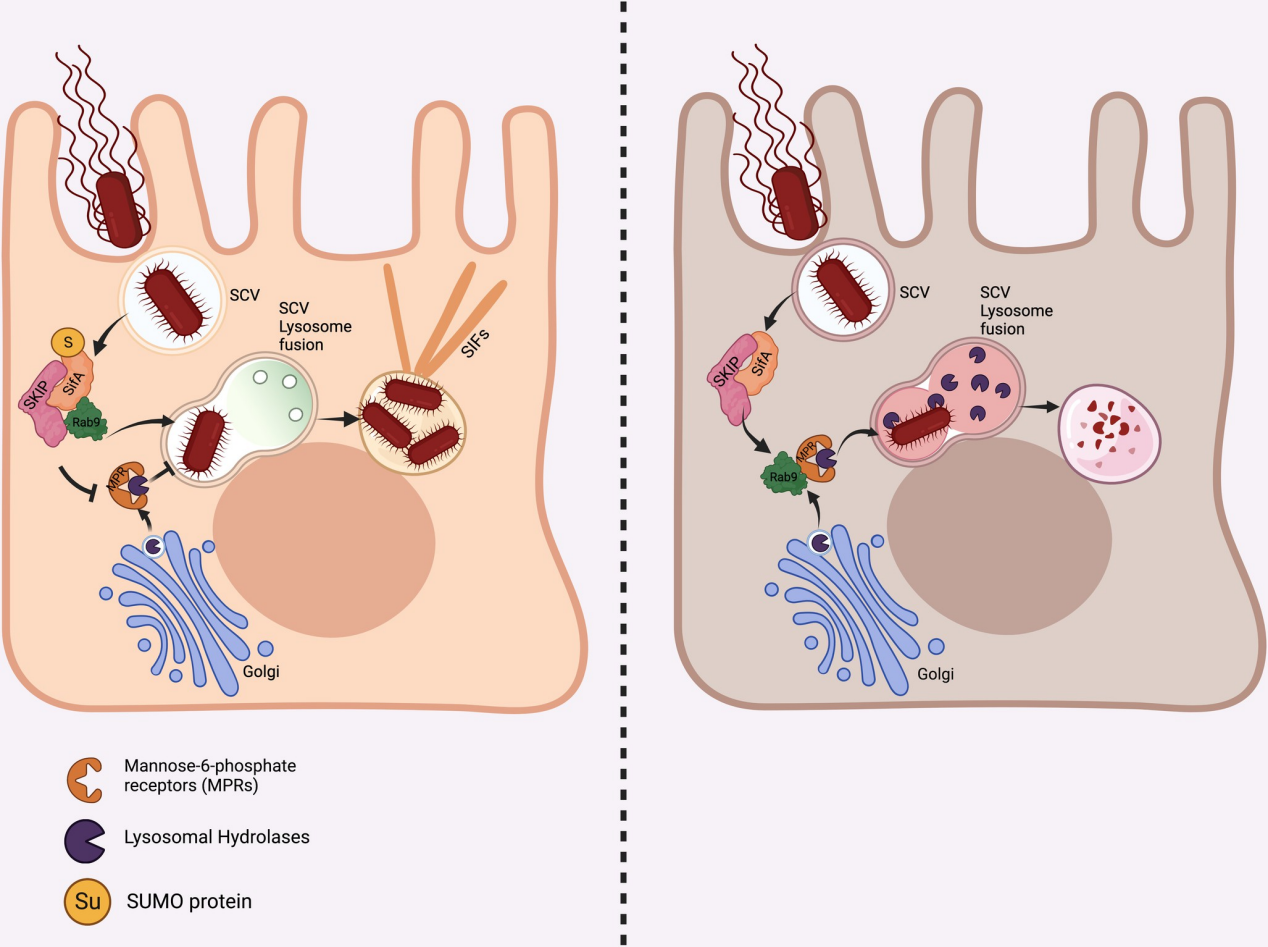
Schematic describing role of SifA SUMOylation in *Salmonella* pathogenesis. SifA SUMOylation is essential for Rab9 sequestration by PLEKHM2 which subverts CI-M6PR routing and thus protects SCV from lysosomal hydrolases. SifA^K11R^ cannot alter this trafficking route which leads to SCV degradation and compromised replication. *This figure has been created using biorender software (**https*:*//biorender*.*com/*)*)*.

Hereby, we concluded that SUMOylation of SifA during infection is essential for *Salmonella* to thrive in the host, overcoming lysosomal attack and dissemination. A schematic of events associated during pathogenesis of *Salmonella* with and without SUMOylation of SifA is represented in Fig 7.

## Discussion

The present study emphasizes a novel regulatory mechanism, where the effector SifA is seen to undergo SUMOylation, a host PTM mechanism, for the progression of Salmonellosis. SPI-2 effector SifA is required for dissemination of *Salmonella* and establishing systemic infection in mice [17, 60]. At the cellular level, SifA is responsible for SCV maintenance and SIF generation, two phenotypic hallmarks associated with the virulence of *Salmonella* [14, 23]. Mechanistically, the presence of SifA subverts trafficking of Rab9-M6PR mediated lysosomal hydrolases targeting SCVs, thereby protecting it from lysosomal degradation [32]. To perform these numerous functions, SifA interacts directly or indirectly with multiple host adaptors including SKIP, PLEKHM1, Rab7, Rab9, LAMP1 simultaneously associating with few other SPI-2 effectors [15, 26, 61, 62]. In this given scenario, the imperative question that needs to be addressed is the factors regulating such strategic placements of these proteins within the complex network. PTM mechanisms are tools regulating almost entire cellular events, which have been shown to be targeted by pathogenic effectors to steer control of host cellular physiology. As reported earlier, SifA undergoes PTMs prenylation and S-acylation for membrane anchorage and achieve destined localization in infected cells [63]. Our group reported PTM SUMOylation to be drastically altered upon *Salmonella* infection [34]. Besides, it was demonstrated that the intracellular fate of *Salmonella* is significantly impacted by the SUMOylation of GTPase Rab7 [13].

Further, other relating studies have shown effectors of various pathogens e.g., AmpA from *Anaplasma* [38] and TRP120 from *E*. *chaffeensis* [39] getting SUMOylated during infection for aiding the pathogen survival inside hosts. Hence, the strong background from all these studies and a need for convincing hypothesis to provide insights regarding functional regulation of SifA paved the foundation of this study.

The current study identified SifA undergoing SUMOylation at its 11^th^ lysine, and further proved how *Salmonella* exploited this modification for its benefit. It is well known that through its interaction with SKIP, SifA contribute to the regulation kinesin-1 mediated anterograde movement of late endosomal compartments [64]. The functions of SUMOylation are well explored in processes like transcriptional regulation, DNA repair, sub cellular localization, substrate folding etc., [65–68] nonetheless their role in regulation of cytoskeletal elements is currently surfacing. Recent studies have revealed that actin gets SUMOylated for nuclear transport [69], also alpha tubulin undergoes SUMO modification to exert control over microtubule assembly [70]. One important aspect associated with control of cytoskeletal proteins is cellular trafficking. SUMOylation enable their substrate cargoes to be transported in both anterograde and retrograde directions reversibly. A study from literature concerning RNA binding La chaperone protein demonstrated that directionality of transport is dependent on SUMOylation at lysine 41 in neuronal axons [71].

Hence, we presume that SifA SUMOylation provides a scaffold to carry out various cellular trafficking that decide the fate of bacterial existence in host cells. Misrouting Rab9 conjugated MPR vesicles preventing SCV acidification, [32],utilizing PLEKHM1 Rab7 complex for mobilizing phagolysosomal membranes for SCV generation, [15, 62] binding to SKIP Kinesin-1 complex for SIF extension [61] are few of the cellular transport orchestrated by SifA. SifA SUMOylation supposedly imparts control to these complex trafficking events during pathogenesis. The host SUMO ligases and deSUMOylases involved in maintenance of SifA SUMOylation and the spatiotemporal regulation of above stated events directed by SUMO are promising fields for future investigations. Nevertheless, our work indicates SUMO is integral component of functional SifA and hampering it collapses the prominent phagolysosomal alterations brought by SifA during pathogenesis. Moreover, the present work cites the first evidence of a *Salmonella* effector undergoing SUMOylation for sustenance inside hosts and discloses additional layers of events in host pathogen cross-talk.

## Materials and methods

### Ethics statement

All experiments included in this manuscript were performed after obtaining due approvals from Institutional Bio Safety Committee (IBSC). Animal experiments were also cleared through Institutional Animal Ethics Committee (IAEC), under the approval numbers- RCB/IAEC/2017/019 & RCB/IAEC/2021/087.

### Cell culture and treatments

Human adenocarcinoma Cell lines HCT-8, were cultured in Rosewell Park Memorial Institute (RPMI) media supplemented with 14 mMNaHCO3 (Sigma), 15 mM HEPES buffer (pH 7.4), 1 mM sodium pyruvate (GIBCO), 40 mg/l penicillin (GIBCO), 90 mg/l streptomycin (GIBCO), and 10% fetal bovine serum (FBS) (GIBCO). Human colorectal adenocarcinoma cell line CaCo2 and human cervical carcinoma cell line HeLa, were grown in Dulbecco’s modified Eagle’s medium (DMEM) comprising14 mM NaHCO3, 15 mM HEPES buffer (pH 7.5), 40 mg/l penicillin, 90 mg/l streptomycin, and 10% FBS. Bone Marrow Derived Macrophages (BMDMs) were prepared from the femur of C57BL/6 mice by flushing them aseptically in DMEM. Cells thus obtained were first treated with RBC lysis buffer (Sigma) and then cultured and differentiated in DMEM supplemented with10 mM HEPES, non-essential amino acids (GIBCO), 10% FBS and 20% L929 conditioned media. The pharmacological inhibitors included in this study include cycloheximide used at a concentration of 100 ug/ml till the indicated time, proteasomal inhibitor MG132 at 20 μM working concentration for a total duration of 4 hours. Vacuolar ATPase inhibitor bafilomycin A1 treatment was given at a concentration of 100 nM for total duration of 4 hours.

### Bacterial Strains, Plasmids, and *In vitro* infection

*Salmonella* Typhimurium strain SL1344 was used throughout the studies. The SifA deletion knockout strain (ΔSifA) in SL1344 background together with the parent strain was kindly gifted by Beth McCormick, University of Massachusetts Medical School, MA. The coding sequence of SifA amplified from SL1344 genome were cloned in a series of prokaryotic expression vectors including pBH-HA from Roche, pET28a, pET21a, pACYC-HA and pGEX-6p1 vectors for various experiments. SUMO intolerant SifA (SifA K11R) was generated via site directed mutagenesis (SDM) in all these above vectors. Plasmids bearing wild type and mutant clones were transformed into the ΔSifA strain via electroporation for the generation of complemented strains ΔSifA/pSifA^WT^ and ΔSifA/pSifA^K11R^ respectively. All the mentioned bacterial strains were cultured in Luria Bertani (LB) broth at 37°C aerobically for 8 hours serving as the primary culture. A secondary culture grown by inoculating the primary culture in 12 ml LB broth in standing white cap tubes (round bottom polypropylene dual position snap cap tubes) under stationary hypoxic conditions overnight at 37°C were used to infect epithelial cells HCT-8, HeLa and Caco2 at a multiplicity of infection (MOI) of 1:40. BMDMs were infected similarly at a MOI of 1:10. Mammalian vector bearing SifA cloned in peGFP-C1 vector was used for mammalian over expression studies.

### Cell transfection

Transfection studies were conducted in HCT-8 and HeLa cells by the standard transfection protocol employing reagents lipofectamine 2000 and lipofectamine LTX (Invitrogen, USA) respectively. Day prior to transfection, 2.5×10^5^ cells were plated in 24-well plates to obtain 80% confluency. Briefly, 1μg of required plasmid and the transfectant reagent taken at a ratio of 1μg plasmid: 2 μl of reagent were separately diluted in minimum serum media Opti-MEM (Invitrogen, USA) and incubated for 5 minutes. The two mixtures were then mixed and incubated for another 15 minutes at room temperature and subsequently supplied to cells growing in Opti-MEM followed by an incubation of 24 hours, after which cells were replenished with complete media.

### Yeast two hybrid assay

The bait fusion construct in vector pGBKT7 expressing GAL4 DNA binding domain (BD) and prey fusion construct in vector pGADT7 expressing GAL4 activation domain (AD) were co-transformed in yeast strain (Y187, Clonetech’s matchmaker Gold Y2H system). Transformants grown on SD media plates devoid of auxotrophic markers leucine and tryptophan at 30°C were replated on selection plate further lacking histidine and adenine to monitor bait prey interaction.

### Protein Purification and *In vitro* SUMOylation assay

*E*. *coli* expression vector pGEX6P1 encoding SifA^WT^ or SifA^K11R^ were transformed in strain BL21 followed by IPTG induction at a concentration 0.05 mM at 37°C. The fusion construct GST SifA^WT^ or GST SifA^K11R^ were purified by using glutathione sepharose beads (GE Healthcare) and further cleaved with thrombin for tag separation. Proteins further purified by chromatography using superdox 200 columns were subjected to SUMO conjugation reactions with the help of commercially available *in vitro* SUMOylation assay kit (Enzo Life Sciences). Kit components comprising SUMO machinery enzymes and ATP were added to 250 nM of wild type SifA and K11R SifA proteins separately in a 20 μl reaction mix along with provided controls at 37°C for 1 hour as per manufacturer’s instructions. The reactions were stopped and processed by adding Laemmle buffer after which it was loaded onto SDS-PAGE and immunoblotted to be probed by SUMO-1 and SUMO2 antibodies.

### In-bacto SUMO Conjugation assay

This assay again included co-expressing SifA^WT^ or SifA^K11R^ from pGEX6P1 vector in *E*. *coli* BL21 along with plasmids encoding SUMO machinery enzymes pE1E2S1 (SUMO-1) or pE1E2S2 (SUMO-2) for the overproduction of SUMO conjugated substrates in bacteria [47]. Rab7 was chosen as positive control for this assay [13]. Double transformants growing on plates supplied with ampicillin and chloramphenicol were cultured in LB in presence of 0.05 mM IPTG for induction at 37°C for 3 hours which is followed by lysates preparation and analysis by western blot.

### Immunoblotting

Cells lysates were prepared in Radioimmunoprecipitation assay buffer (RIPA) (Sigma) supplemented with Protease Inhibitor Cocktail (G biosciences). These lysates were further denatured by boiling in 2x SDS Laemelli buffer (20 mM Tris-HCl pH 8.0, 150 mM KCl,10% glycerol, 5 mM MgCl2 and 0.1% NP40) at 95°C for 10 minutes. CBX protein assaykit (G-Bioscience, USA) was utilized for protein quantification. Lysates were then separated by routine sodium dodecyl sulfate–polyacrylamide gel electrophoresis (SDS-PAGE) in tris glycine running buffer. Resolved gels were transferred on to nitrocellulose membrane (BioRad) after which the blots were probed with antibodies against SUMO-1 (CST), SUMO-2 (CST), GST (Sigma), eGFP (Abcam), PLEKHM2 (Abcam), CI-M6PR (Abcam) Actin (Thermo Scientific), and GAPDH (Invitrogen). The instrument ImageQuant LAS4000 from GE was used for blot developing and processing and densitometry analysis was done using the Image J software.

### Immunoprecipitation

For immunoprecipitation HCT-8 cells were lysed in non-denaturation immunoprecipitation lysis buffer (Pierce, Thermo Scientific) supplemented with 20 mM N-Ethyl Maleimide (NEM), (Sigma) and 2x protease inhibitor cocktail (G biosciences) for 20minutes on incubation on ice. Lysates were prepared by centrifugation at 15700 g for 10 minutes at 4°C and collecting the supernatant. The lysates obtained were subjected to preclearing by incubation with protein G sepharose beads (GE) for 30 minutes at 4°C on an end-to-end rotor. Precleared lysates after centrifugation were then incubated with anti-SUMO1 antibody at 4°C on an end-to-end rotor for overnight. Next day, the incubated lysates were precipitated by addition of protein G sepharose beads for 3hours at 4°C. Precipitations were also carried by IgG raised in same host (Merck Millipore) as the isotype control. The antigen-antibody complex obtained after washing with lysis buffer was processed in Laemmle buffer for immunoblotting.

### Immunofluorescence

HeLa and HCT-8 cells were grown on cover slips embedded in 24-well plates. In assays monitoring lysosomal activity ratiometric probes like DQ-BSA, lysotracker and lysosensor were incorporated in cells prior to fixation as per manufacturer’s instructions. Post infection, cellswere washed thrice in 1× phosphate buffered saline (PBS) and fixed in 4% methanol free paraformaldehyde (CST) for 15 min at room temperature (RT). Following washing, cells were permeabilized in 0.5% saponin in PBS for 10 minutes at RT after which they were blocked in blocking solution (1%BSA in PBS containing 0.1% Tween 20) for 1 hour at RT. Cells were incubated in primary antibodies against LAMP1 (Sigma), Rab9 (Invitrogen), GFP (1:200), Galectin-8 (Abcam), CI-M6PR (Abcam), SUMO-1(Invitrogen), *Salmonella* LPS (Abcam) prepared in blocking solution for overnight at 4°C. Next day, cells were washed four times in PBS and proceeded for incubation in fluorophore conjugated secondary antibodies for 2 hours at RT in PBS containing 0.5% BSA and 0.1% Tween-20. Cells were finally washed three times and incubated in 4,6-diamidino-2-phenylindole (DAPI) (1 μg/ml; Sigma-Aldrich) for 5 min in dark. Mounting was done using prolong diamond antifade (Invitrogen). Images were acquired in Leica SP8 confocal microscopy under 63 × oil immersion objectives. Co-localization analysis and intensity calculations were obtained from acquired images using SP-8 LasX software and Image J.

### Mass spectrometry

*In vitro* SUMOylated purified SifA samples were run on normal SDS PAGE gel (5% stacking, 12% resolving), stained in CBB staining solution (0.1% Coomassie Brilliant Blue R-250, 50% methanol and 10% glacial acetic acid) followed by destaining in solution (40% methanol and 10% glacial acetic acid). Samples were excised with a clean scalpel into finer slices and taken for further processing. The gel slices were further destained with treatment of 50% ammonium bicarbonate and 50mM acetonitrile solution mix. The destained gel slices are subjected to trypsin and chymotrypsin digestion in the ratio of 1:20 (1μg trypsin for 20 μg protein) and left for 16hours at 37-degree Celsius. Next day the peptides are extracted in 50% ACN and proteome grade water containing 0.1% formic acid. The peptides were concentrated in a vacuum drier, treated with ZipTip (Thermo) for salts and SDS removal and handed over for injection in mass spectrometer (ab sciex 6500). The peptides obtained were searched against uniprot ID of SifA (E8XFF1-SALT4) for specific modifications.

### Proximity ligation assay

Proximity Ligation Assay (PLA) was conducted using Duo-link PLA kit (Sigma) as per manufacturer’s protocol. Cells after permeabilization as described before were blocked in PLA blocking buffer followed by incubation in primary antibody against GFP (rabbit) and SUMO-1(mouse) overnight. Next day, the coverslips were washed in buffer A provided in the kit. Further, the samples were incubated with plus and minus end PLA probes supplied by the manufacturer. Hereafter the attached probes were ligated and subsequently PCR amplified for 100 minutes using the provided reagents. All the reactions were performed at 37°C in a humidity chamber. Finally, the samples were washed in buffer B and mounted onto a slide with the supplied mountant containing DAPI for visualization. The PLA signal punctae were quantified from indicated no. of cells across each sample and plotted using the graph pad software.

### Gentamycin protection assay

Infection in cells by various strains at an MOI of 1:40 in medium lacking pen strep were allowed to proceed for 1 hour at 37°C in the incubator, followed by treatment with antibiotic Gentamycin (Gibco) at a higher concentration of 100 μg/ml in media for 1 hour to eradicate extracellular bacteria. After this step, cells were washed in sterile PBS followed by further incubation in the media containing gentamycin at a concentration of 20 μg/ml for the rest of the infection. At the end, infected cells were lysed using PBS containing 0.1% triton X-100 detergent followed by dilution plating on LB plates added with streptomycin (50 μg/ml). The plates were incubated in a 37°C incubator and individual CFU were counted and graphically represented.

### Mice infections

All in vivo studies were conducted in C57BL/6 female mice (6–8 weeks). All the animal experiments were carried out in the Small Animal Facility of Regional Centre for Biotechnology (RCB). The experiments were performed after approval by the RCB Institutional Animal Ethics Committee (approval no. RCB/IAEC/2017/019 & RCB/IAEC/2021/087). For the induction of colitis streptomycin pre-treated colitis model was employed [72]. Food and water were removed 4 hours prior to infection followed by a treatment with 20 mg/Kg of streptomycin by oral gavage for the clearance of microbiota. After 24 hours, *Salmonella* SL1344 strain at a MOI of 5x10^7^ CFU per mice was fed using oral gavage after 4 hours of withdrawal of food and water. Post 48 hours of infection, mice were euthanized and various organs including colon, spleen, MLNs, and faecal pellets were harvested. The tissues from organs were homogenized in PBS+ 0.1% Triton-X solution in Precelly’s bead beater followed by dilution plating on McConkey agar plates containing streptomycin (100 μg/ml) for bacterial enumeration. For the survival assay, mice infected by the mentioned strains as described above were left monitored for death succumbing to progression of infection. A survival curve indicating the no. of days each mouse survived across all groups were plotted using graph pad prism software.

### Competitive index assay

This assay involves infecting the same mice with mixed inocula comprising both wild type and mutant protein expressing strains in equal ratio [59, 73]. The strains ΔSifA/pSifA^WT^ (strain1) ΔSifA/pSifA^K11R^ (strain2) were cloned in pET28a vector (Kan resistance) and pET21a vector (Amp resistance) respectively, thus enabling a plasmid-based antibiotic marker differentiation. The input bacterial inoculum containing equal concentrations of both strain 1 and strain 2 totaling to a MOI of 5x10^7^ CFU standardized by absorbance based CFU determination were fed to the C57BL/6 mice as described earlier. Homogenized tissue lysates from organs collected post 48 hours of infection were simultaneously plated on separate McConkey agar strep plates supplied with kanamycin (50 μg/ml) or ampicillin (100 μg/ml). CI was calculated as the CFU of mutant-to-wild-type ratio obtained from target sample, divided by the corresponding CFU ratio in the inoculum (input sample).

### Haematoxylin and Eosin staining

Proximal colon sections were fixed in 10% formalin buffer for two days at room temperature and frozen in holders with a thick layer of cryomatrix (Thermo Scientific) and stored at -80°C till use. Five micrometre thick sections were cut onto glass slides in a microtome and processed for haematoxylin (Sigma) and eosin (Sigma) staining. The slides and mounted using DPX mountant (Sigma) and bright field images were taken using a Nikon (NY, USA) inverted fluorescence microscope.

### Statistics

The results are expressed as the mean standard error from an individual experiment done in triplicate. Data were analyzed either with one way ANOVA or by standard unpaired two-tailed Student’s t test, with *p* values of 0.05–0.001 considered statistically significant. All graphs and stats were plotted using the graph pad prism software.

## Supporting information

S1 Fig **(A)** Co-immunoprecipitation of SUMOylated SifA from HCT-8 cells during infection by complemented strains pSifA^WT^ and pSifA^K11R^ expressing SifA-HA. IP was carried from infected protein lysates 7 hours post infection by SUMO-1 antibody followed by immunoblotting by anti-HA antibody. A faint band corresponding to SUMOylated SifA shown was obtained only in pSifA^WT^ lane. This is a representative blot of single experiment. **(B)** Images displaying discrete PLA punctae representing co-localization of SifA and SUMO-1 in indicated samples (scale 6 micron). **(C)** Quantification of PLA punctae carried out using particle analysis tool from Image J software.
(TIF)

S2 Fig **(A)** Timeline of expression levels of ectopically expressed SifA^WT^ and SifA^K11R^ proteins from HCT-8 cell lysates. **(B)** Lysates prepared after 48 hours of transfection, showed a significant change in expression. **(C)** Live cell snapshots of transfected HCT-8 cells indicating localization of GFP SifA^WT^, GFP SifA^K11R^, and empty vector with lysosomes labeled by lysotracker red (scale 8 microns). Corrected image sum intensity quantified for **(D)** GFP and **(E)** lysotracker red fluorescence. **(F)** Co-localization analysis between both channels (GFP and lysotracker) for all three transfected conditions are also performed. The blots shown here are representative of at least three biological replicates.
(TIF)

S3 Fig **(A)** Images co-stained for Rab9 and CI-M6PR for co-localization analysis in HeLa cells under pSifA^WT^ (scale 9 micron) and pSifA^K11R^ (scale 11 microns) infection conditions. **(B)** Distribution and quantization of CI-M6PR over SCVs from similar infection conditions (scale 10 microns). SCV marker LAMP1 was used to indicate SCVs. *Salmonella* used here expresses mCherry plasmid for visualization.
(TIF)

S4 Fig **(A)** Images co-stained for Rab9 and LAMP1 to analyze Rab9 co-localization over SCVs from infected HeLa cells by depicted strains expressing mCherry (scale 10 microns). **(B)** Quantification of co-localization of Rab9 over SCVs from acquired images.
(TIF)

S5 Fig **(A)** The expression levels of SifA^WT^ and SifA ^K11R^ proteins from ΔSifA complemented with SifA^WT^ (SifA^WT^) and SifA^K11R^ (SifA^K11R^) respectively. (**B**) The growth curve indicating division rate of strains SL1344, ΔSifA, SifA^WT^ and SifA^K11R^. Comparison of CFU results obtained from SifA^WT^ and SifA^KIIR^ infected **(C)** HeLa **(D)** Caco2 cells and **(E)** RAW 264.7cells post 16 hours of infection. **(F)** Interaction of transfected GFP SifA with ubiquitin from HCT-8 cells. SifA is shown to be ubiquitinated at lysine 11 by co-IP assay using GFP antibody, followed by immunoblotting using both ubiquitin and GFP antibodies. **(G)** Expression levels of Ubc-9 in presence of scrambled and Ubc-9 siRNA. **(H)** Replication rates of SifA^WT^ and SifA^K11R^ strains under Ubc-9 knockdown conditions. The blots shown here are representative of at least three biological replicates.
(TIF)

S1 Data SUMOylation reaction conducted with purified SifA was subjected to mass spectrometry for validating whether lysine 11 undergoes the SUMO-modification (Fig 1H). The additional data (S1 Data) provides the list of peptides obtained from mass spectrometry search conducted. The peptide bearing diglycine of SUMO conjugated at 11^th^ lysine is highlighted in the file.
(XLSX)

## References

[1] Majowicz SE, Musto J, Scallan E, Angulo FJ, Kirk M, O’Brien SJ, et al. The global burden of nontyphoidal Salmonella gastroenteritis. Clin Infect Dis. 2010;50:882–9. doi: 10.1086/650733 20158401

[2] Stanaway JD, Parisi A, Sarkar K, Blacker BF, Reiner RC, Hay SI, et al. The global burden of non-typhoidal salmonella invasive disease: a systematic analysis for the Global Burden of Disease Study 2017. The Lancet Infectious Diseases [Internet]. 2019 [cited 2023 Jul 12];19:1312–24. Available from: https://www.thelancet.com/journals/laninf/article/PIIS1473-3099(19)30418-9/fulltext. doi: 10.1016/S1473-3099(19)30418-9 31562022 PMC6892270

[3] Ke Y, Lu W, Liu W, Zhu P, Chen Q, Zhu Z. Non-typhoidal Salmonella infections among children in a tertiary hospital in Ningbo, Zhejiang, China, 2012–2019. PLOS Neglected Tropical Diseases [Internet]. 2020 [cited 2023 Jul 13];14:e0008732. Available from: https://journals.plos.org/plosntds/article?id=10.1371/journal.pntd.0008732. doi: 10.1371/journal.pntd.0008732 33017418 PMC7561262

[4] Marcus SL, Brumell JH, Pfeifer CG, Finlay BB. Salmonella pathogenicity islands: big virulence in small packages. Microbes Infect. 2000;2:145–56. doi: 10.1016/s1286-4579(00)00273-2 10742687

[5] McGhie EJ, Hayward RD, Koronakis V. Control of actin turnover by a salmonella invasion protein. Mol Cell. 2004;13:497–510. doi: 10.1016/s1097-2765(04)00053-x 14992720

[6] Lou L, Zhang P, Piao R, Wang Y. Salmonella Pathogenicity Island 1 (SPI-1) and Its Complex Regulatory Network. Front Cell Infect Microbiol [Internet]. 2019 [cited 2022 Nov 29];9:270. Available from: https://www.ncbi.nlm.nih.gov/pmc/articles/PMC6689963/. doi: 10.3389/fcimb.2019.00270 31428589 PMC6689963

[7] Steele-Mortimer O. The Salmonella-containing Vacuole–Moving with the Times. Curr Opin Microbiol [Internet]. 2008 [cited 2023 Jan 15];11:38–45. Available from: https://www.ncbi.nlm.nih.gov/pmc/articles/PMC2577838/. doi: 10.1016/j.mib.2008.01.002 18304858 PMC2577838

[8] Birmingham CL, Brumell JH. Autophagy recognizes intracellular Salmonella enterica serovar Typhimurium in damaged vacuoles. Autophagy. 2006;2:156–8. doi: 10.4161/auto.2825 16874057

[9] Birmingham CL, Smith AC, Bakowski MA, Yoshimori T, Brumell JH. Autophagy Controls Salmonella Infection in Response to Damage to the Salmonella-containing Vacuole*. Journal of Biological Chemistry [Internet]. 2006 [cited 2023 Jan 15];281:11374–83. Available from: https://www.sciencedirect.com/science/article/pii/S0021925819563153. doi: 10.1074/jbc.M509157200 16495224

[10] Thurston TLM, Wandel MP, von Muhlinen N, Foeglein Á, Randow F. Galectin-8 targets damaged vesicles for autophagy to defend cells against bacterial invasion. Nature [Internet]. 2012 [cited 2023 Jan 16];482:414–8. Available from: https://www.ncbi.nlm.nih.gov/pmc/articles/PMC3343631/. doi: 10.1038/nature10744 22246324 PMC3343631

[11] Smith AC, Heo WD, Braun V, Jiang X, Macrae C, Casanova JE, et al. A network of Rab GTPases controls phagosome maturation and is modulated by Salmonella enterica serovar Typhimurium. J Cell Biol. 2007;176:263–8. doi: 10.1083/jcb.200611056 17261845 PMC2063952

[12] Méresse S, Steele-Mortimer O, Finlay BB, Gorvel JP. The rab7 GTPase controls the maturation of Salmonella typhimurium-containing vacuoles in HeLa cells. EMBO J [Internet]. 1999 [cited 2023 Jan 16];18:4394–403. Available from: https://www.ncbi.nlm.nih.gov/pmc/articles/PMC1171514/. doi: 10.1093/emboj/18.16.4394 10449405 PMC1171514

[13] Mohapatra G, Gaur P, Mujagond P, Singh M, Rana S, Pratap S, et al. A SUMOylation-dependent switch of RAB7 governs intracellular life and pathogenesis of Salmonella Typhimurium. J Cell Sci. 2019;132:jcs222612. doi: 10.1242/jcs.222612 30510112

[14] Beuzón CR, Méresse S, Unsworth KE, Ruíz-Albert J, Garvis S, Waterman SR, et al. Salmonella maintains the integrity of its intracellular vacuole through the action of SifA. EMBO J [Internet]. 2000 [cited 2023 Jan 15];19:3235–49. Available from: https://www.ncbi.nlm.nih.gov/pmc/articles/PMC313946/. doi: 10.1093/emboj/19.13.3235 10880437 PMC313946

[15] McEwan DG, Richter B, Claudi B, Wigge C, Wild P, Farhan H, et al. PLEKHM1 regulates Salmonella-containing vacuole biogenesis and infection. Cell Host Microbe. 2015;17:58–71. doi: 10.1016/j.chom.2014.11.011 25500191

[16] Rawet-Slobodkin M, Elazar Z. PLEKHM1: A Multiprotein Adaptor for the Endolysosomal System. Molecular Cell [Internet]. 2015 [cited 2023 Jan 15];57:1–3. Available from: https://www.cell.com/molecular-cell/abstract/S1097-2765(14)01000-4. doi: 10.1016/j.molcel.2014.12.022 25574946

[17] Brumell JH, Rosenberger CM, Gotto GT, Marcus SL, Finlay BB. SifA permits survival and replication of Salmonella typhimurium in murine macrophages. Cellular Microbiology [Internet]. 2001 [cited 2023 Jan 15];3:75–84. Available from: https://onlinelibrary.wiley.com/doi/abs/ doi: 10.1046/j.1462-5822.2001.00087.x 11207622

[18] Drecktrah D, Levine-Wilkinson S, Dam T, Winfree S, Knodler LA, Schroer TA, et al. Dynamic behavior of Salmonella-induced membrane tubules in epithelial cells. Traffic. 2008;9:2117–29. doi: 10.1111/j.1600-0854.2008.00830.x 18785994 PMC2682622

[19] Rajashekar R, Liebl D, Seitz A, Hensel M. Dynamic remodeling of the endosomal system during formation of Salmonella-induced filaments by intracellular Salmonella enterica. Traffic. 2008;9:2100–16. doi: 10.1111/j.1600-0854.2008.00821.x 18817527

[20] Kuhle V, Abrahams GL, Hensel M. Intracellular Salmonella enterica redirect exocytic transport processes in a Salmonella pathogenicity island 2-dependent manner. Traffic. 2006;7:716–30. doi: 10.1111/j.1600-0854.2006.00422.x 16637890

[21] Zhang Y, Hensel M. Evaluation of nanoparticles as endocytic tracers in cellular microbiology. Nanoscale [Internet]. 2013 [cited 2023 Jan 15];5:9296–309. Available from: https://pubs.rsc.org/en/content/articlelanding/2013/nr/c3nr01550e. doi: 10.1039/c3nr01550e 23942623

[22] Liss V, Swart AL, Kehl A, Hermanns N, Zhang Y, Chikkaballi D, et al. Salmonella enterica Remodels the Host Cell Endosomal System for Efficient Intravacuolar Nutrition. Cell Host Microbe. 2017;21:390–402. doi: 10.1016/j.chom.2017.02.005 28238623

[23] Brumell JH, Goosney DL, Finlay BB. SifA, a type III secreted effector of Salmonella typhimurium, directs Salmonella-induced filament (Sif) formation along microtubules. Traffic. 2002;3:407–15. doi: 10.1034/j.1600-0854.2002.30604.x 12010459

[24] Garcia-del Portillo F, Zwick MB, Leung KY, Finlay BB. Salmonella induces the formation of filamentous structures containing lysosomal membrane glycoproteins in epithelial cells. Proc Natl Acad Sci U S A. 1993;90:10544–8. doi: 10.1073/pnas.90.22.10544 8248143 PMC47813

[25] Stein MA, Leung KY, Zwick M, Garcia-del Portillo F, Finlay BB. Identification of a Salmonella virulence gene required for formation of filamentous structures containing lysosomal membrane glycoproteins within epithelial cells. Mol Microbiol. 1996;20:151–64. doi: 10.1111/j.1365-2958.1996.tb02497.x 8861213

[26] Diacovich L, Dumont A, Lafitte D, Soprano E, Guilhon A-A, Bignon C, et al. Interaction between the SifA Virulence Factor and Its Host Target SKIP Is Essential for Salmonella Pathogenesis. The Journal of Biological Chemistry [Internet]. 2009 [cited 2023 Jan 16];284:33151. Available from: https://www.ncbi.nlm.nih.gov/pmc/articles/PMC2785157/. doi: 10.1074/jbc.M109.034975 19801640 PMC2785157

[27] Fang Z, Fallet M, Moest T, Gorvel J-P, Méresse S. The Salmonella effector SifA initiates a kinesin-1 and kinesin-3 recruitment process mirroring that mediated by Arl8a and Arl8b. J Cell Sci. 2022;135:jcs259183. doi: 10.1242/jcs.259183 34878110

[28] Boucrot E, Henry T, Borg J-P, Gorvel J-P, Méresse S. The Intracellular Fate of Salmonella Depends on the Recruitment of Kinesin. Science [Internet]. 2005 [cited 2023 Jan 16];308:1174–8. Available from: https://www.science.org/doi/abs/10.1126/science.1110225. 15905402 10.1126/science.1110225

[29] Garcia-del Portillo F, Finlay BB. Targeting of Salmonella typhimurium to vesicles containing lysosomal membrane glycoproteins bypasses compartments with mannose 6-phosphate receptors. J Cell Biol. 1995;129:81–97. doi: 10.1083/jcb.129.1.81 7698996 PMC2120372

[30] Rathman M, Barker LP, Falkow S. The unique trafficking pattern of Salmonella typhimurium-containing phagosomes in murine macrophages is independent of the mechanism of bacterial entry. Infect Immun. 1997;65:1475–85. doi: 10.1128/iai.65.4.1475-1485.1997 9119490 PMC175156

[31] Barbero P, Bittova L, Pfeffer SR. Visualization of Rab9-mediated vesicle transport from endosomes to the trans-Golgi in living cells. J Cell Biol [Internet]. 2002 [cited 2023 Jan 16];156:511–8. Available from: https://www.ncbi.nlm.nih.gov/pmc/articles/PMC2173336/. doi: 10.1083/jcb.200109030 11827983 PMC2173336

[32] McGourty K, Thurston TL, Matthews SA, Pinaud L, Mota LJ, Holden DW. Salmonella Inhibits Retrograde Trafficking of Mannose-6-Phosphate Receptors and Lysosome Function. Science [Internet]. 2012 [cited 2023 Jan 16];338:963–7. Available from: https://www.ncbi.nlm.nih.gov/pmc/articles/PMC6485626/. doi: 10.1126/science.1227037 23162002 PMC6485626

[33] Jackson LK, Nawabi P, Hentea C, Roark EA, Haldar K. The Salmonella virulence protein SifA is a G protein antagonist. Proceedings of the National Academy of Sciences [Internet]. 2008 [cited 2023 Jan 16];105:14141–6. Available from: https://www.pnas.org/doi/10.1073/pnas.0801872105. 18787122 10.1073/pnas.0801872105 PMC2544592

[34] Verma S, Mohapatra G, Ahmad SM, Rana S, Jain S, Khalsa JK, et al. Salmonella Engages Host MicroRNAs To Modulate SUMOylation: a New Arsenal for Intracellular Survival. Mol Cell Biol [Internet]. 2015 [cited 2023 Jan 16];35:2932–46. Available from: https://www.ncbi.nlm.nih.gov/pmc/articles/PMC4525320/. doi: 10.1128/MCB.00397-15 26100020 PMC4525320

[35] Wilkinson KA, Henley JM. Mechanisms, regulation and consequences of protein SUMOylation. Biochem J [Internet]. 2010 [cited 2023 Jan 16];428:133–45. Available from: https://www.ncbi.nlm.nih.gov/pmc/articles/PMC3310159/. doi: 10.1042/BJ20100158 20462400 PMC3310159

[36] Saitoh H, Hinchey J. Functional Heterogeneity of Small Ubiquitin-related Protein Modifiers SUMO-1 versus SUMO-2/3*. Journal of Biological Chemistry [Internet]. 2000 [cited 2023 Jul 13];275:6252–8. Available from: https://www.sciencedirect.com/science/article/pii/S0021925818304794. doi: 10.1074/jbc.275.9.6252 10692421

[37] Ulrich HD. The Fast-Growing Business of SUMO Chains. Molecular Cell [Internet]. 2008 [cited 2023 Sep 19];32:301–5. Available from: https://www.cell.com/molecular-cell/abstract/S1097-2765(08)00697-7. doi: 10.1016/j.molcel.2008.10.010 18995828

[38] Beyer AR, Truchan HK, May LJ, Walker NJ, Borjesson DL, Carlyon JA. The Anaplasma phagocytophilum effector AmpA hijacks host cell SUMOylation. Cell Microbiol. 2015;17:504–19. doi: 10.1111/cmi.12380 25308709 PMC4664186

[39] Dunphy PS, Luo T, McBride JW. Ehrlichia chaffeensis Exploits Host SUMOylation Pathways To Mediate Effector-Host Interactions and Promote Intracellular Survival. Infect Immun [Internet]. 2014 [cited 2022 Dec 7];82:4154–68. Available from: https://www.ncbi.nlm.nih.gov/pmc/articles/PMC4187855/. doi: 10.1128/IAI.01984-14 25047847 PMC4187855

[40] Tammsalu T, Matic I, Jaffray EG, Ibrahim AFM, Tatham MH, Hay RT. Proteome-wide Identification of SUMO2 Modification Sites. Sci Signal [Internet]. 2014 [cited 2023 Sep 8];7:rs2. Available from: https://www.ncbi.nlm.nih.gov/pmc/articles/PMC4051997/. doi: 10.1126/scisignal.2005146 24782567 PMC4051997

[41] Celen AB, Sahin U. Sumoylation on its 25th anniversary: mechanisms, pathology, and emerging concepts. The FEBS Journal [Internet]. 2020 [cited 2023 Sep 8];287:3110–40. Available from: https://onlinelibrary.wiley.com/doi/abs/10.1111/febs.15319. 32255256 10.1111/febs.15319

[42] Zhao Q, Xie Y, Zheng Y, Jiang S, Liu W, Mu W, et al. GPS-SUMO: a tool for the prediction of sumoylation sites and SUMO-interaction motifs. Nucleic Acids Res [Internet]. 2014 [cited 2023 Jan 16];42:W325–30. Available from: https://www.ncbi.nlm.nih.gov/pmc/articles/PMC4086084/. doi: 10.1093/nar/gku383 24880689 PMC4086084

[43] Beauclair G, Bridier-Nahmias A, Zagury J-F, Saïb A, Zamborlini A. JASSA: a comprehensive tool for prediction of SUMOylation sites and SIMs. Bioinformatics. 2015;31:3483–91. doi: 10.1093/bioinformatics/btv403 26142185

[44] Sampson DA, Wang M, Matunis MJ. The small ubiquitin-like modifier-1 (SUMO-1) consensus sequence mediates Ubc9 binding and is essential for SUMO-1 modification. J Biol Chem. 2001;276:21664–9. doi: 10.1074/jbc.M100006200 11259410

[45] Lascorz J, Codina-Fabra J, Reverter D, Torres-Rosell J. SUMO-SIM interactions: From structure to biological functions. Semin Cell Dev Biol. 2022;132:193–202. doi: 10.1016/j.semcdb.2021.11.007 34840078

[46] Matunis MJ, Wu J, Blobel G. SUMO-1 Modification and Its Role in Targeting the Ran GTPase-activating Protein, RanGAP1, to the Nuclear Pore Complex. J Cell Biol [Internet]. 1998 [cited 2023 Jul 13];140:499–509. Available from: https://www.ncbi.nlm.nih.gov/pmc/articles/PMC2140169/. doi: 10.1083/jcb.140.3.499 9456312 PMC2140169

[47] Uchimura Y, Nakamura M, Sugasawa K, Nakao M, Saitoh H. Overproduction of eukaryotic SUMO-1- and SUMO-2-conjugated proteins in Escherichia coli. Anal Biochem. 2004;331:204–6. doi: 10.1016/j.ab.2004.04.034 15246018

[48] Kao S-H, Wang W-L, Chen C-Y, Chang Y-L, Wu Y-Y, Wang Y-T, et al. Analysis of Protein Stability by the Cycloheximide Chase Assay. Bio Protoc [Internet]. 2015 [cited 2023 Jan 16];5:e1374. Available from: https://europepmc.org/articles/PMC5659619. doi: 10.21769/BioProtoc.1374 29082276 PMC5659619

[49] Tokumoto T, Yamashita M, Tokumoto M, Katsu Y, Horiguchi R, Kajiura H, et al. Initiation of cyclin B degradation by the 26S proteasome upon egg activation. J Cell Biol. 1997;138:1313–22. doi: 10.1083/jcb.138.6.1313 9298986 PMC2132556

[50] Ikeda H, Kerppola TK. Lysosomal localization of ubiquitinated Jun requires multiple determinants in a lysine-27-linked polyubiquitin conjugate. Mol Biol Cell. 2008;19:4588–601. doi: 10.1091/mbc.e08-05-0496 18716056 PMC2575148

[51] Laopanupong T, Prombutara P, Kanjanasirirat P, Benjaskulluecha S, Boonmee A, Palaga T, et al. Lysosome repositioning as an autophagy escape mechanism by Mycobacterium tuberculosis Beijing strain. Sci Rep [Internet]. 2021 [cited 2023 Jan 16];11:4342. Available from: https://www.ncbi.nlm.nih.gov/pmc/articles/PMC7900199/. doi: 10.1038/s41598-021-83835-4 33619301 PMC7900199

[52] Giannella RA, Washington O, Gemski P, Formal SB. Invasion of HeLa Cells by Salmonella typhimurium: A Model for Study of Invasiveness of Salmonella. The Journal of Infectious Diseases [Internet]. 1973 [cited 2023 Jan 16];128:69–75. Available from: doi: 10.1093/infdis/128.1.69 4577975

[53] Niesel DW, Chambers CE, Stockman SL. Quantitation of HeLa cell monolayer invasion by Shigella and Salmonella species. Journal of Clinical Microbiology [Internet]. 1985 [cited 2023 Jan 16];22:897–902. Available from: https://journals.asm.org/doi/abs/10.1128/jcm.22.6.897-902.1985. 4066921 10.1128/jcm.22.6.897-902.1985 PMC271847

[54] Misselwitz B, Kreibich SK, Rout S, Stecher B, Periaswamy B, Hardt W-D. Salmonella enterica Serovar Typhimurium Binds to HeLa Cells via Fim-Mediated Reversible Adhesion and Irreversible Type Three Secretion System 1-Mediated Docking. Infection and Immunity [Internet]. 2010 [cited 2023 Jan 16]; Available from: https://journals.asm.org/doi/10.1128/IAI.00581-10. 20974826 10.1128/IAI.00581-10 PMC3019867

[55] DePedro HM, Urayama P. Using LysoSensor Yellow/Blue DND-160 to sense acidic pH under high hydrostatic pressures. Anal Biochem. 2009;384:359–61. doi: 10.1016/j.ab.2008.10.007 18976626

[56] Albrecht LV, Tejeda-Muñoz N, De Robertis EM. Protocol for Probing Regulated Lysosomal Activity and Function in Living Cells. STAR Protocols [Internet]. 2020 [cited 2023 Jan 16];1:100132. Available from: https://www.sciencedirect.com/science/article/pii/S2666166720301192. doi: 10.1016/j.xpro.2020.100132 33377026 PMC7757114

[57] Brumell JH, Tang P, Mills SD, Finlay BB. Characterization of Salmonella-Induced Filaments (Sifs) Reveals a Delayed Interaction Between Salmonella-Containing Vacuoles and Late Endocytic Compartments. Traffic [Internet]. 2001 [cited 2023 Jan 16];2:643–53. Available from: https://onlinelibrary.wiley.com/doi/abs/10.1034/j.1600-0854.2001.20907.x. 11555418 10.1034/j.1600-0854.2001.20907.x

[58] Knodler LA. Salmonella enterica: living a double life in epithelial cells. Curr Opin Microbiol. 2015;23:23–31. doi: 10.1016/j.mib.2014.10.010 25461569

[59] Macho AP, Zumaquero A, Ortiz-Martín I, Beuzón CR. Competitive index in mixed infections: a sensitive and accurate assay for the genetic analysis of Pseudomonas syringae–plant interactions. Molecular Plant Pathology [Internet]. 2007 [cited 2023 Jan 16];8:437–50. Available from: https://onlinelibrary.wiley.com/doi/abs/10.1111/j.1364-3703.2007.00404.x. 20507512 10.1111/j.1364-3703.2007.00404.x

[60] Fierer J, Okamoto S, Banerjee A, Guiney DG. Diarrhea and Colitis in Mice Require the Salmonella Pathogenicity Island 2-Encoded Secretion Function but Not SifA or Spv Effectors. Infect Immun [Internet]. 2012 [cited 2023 Jan 16];80:3360–70. Available from: https://www.ncbi.nlm.nih.gov/pmc/articles/PMC3457546/. doi: 10.1128/IAI.00404-12 22778101 PMC3457546

[61] Ohlson MB, Huang Z, Alto NM, Blanc M-P, Dixon JE, Chai J, et al. Structure and function of SifA indicate that interactions with SKIP, SseJ, and RhoA family GTPases induce endosomal tubulation. Cell Host Microbe [Internet]. 2008 [cited 2023 Jan 16];4:434–46. Available from: https://www.ncbi.nlm.nih.gov/pmc/articles/PMC2658612/. 18996344 10.1016/j.chom.2008.08.012 PMC2658612

[62] Sindhwani A, Arya SB, Kaur H, Jagga D, Tuli A, Sharma M. Salmonella exploits the host endolysosomal tethering factor HOPS complex to promote its intravacuolar replication. PLoS Pathog. 2017;13:e1006700. doi: 10.1371/journal.ppat.1006700 29084291 PMC5679646

[63] Reinicke AT, Hutchinson JL, Magee AI, Mastroeni P, Trowsdale J, Kelly AP. A Salmonella typhimurium effector protein SifA is modified by host cell prenylation and S-acylation machinery. J Biol Chem. 2005;280:14620–7. doi: 10.1074/jbc.M500076200 15710609

[64] Dumont A, Boucrot E, Drevensek S, Daire V, Gorvel J-P, Poüs C, et al. SKIP, the host target of the Salmonella virulence factor SifA, promotes kinesin-1-dependent vacuolar membrane exchanges. Traffic. 2010;11:899–911. doi: 10.1111/j.1600-0854.2010.01069.x 20406420

[65] Sarangi P, Zhao X. SUMO-mediated regulation of DNA damage repair and responses. Trends Biochem Sci [Internet]. 2015 [cited 2023 Jan 16];40:233–42. Available from: https://www.ncbi.nlm.nih.gov/pmc/articles/PMC4380773/. doi: 10.1016/j.tibs.2015.02.006 25778614 PMC4380773

[66] Abe J, Sandhu UG, Hoang NM, Thangam M, Quintana-Quezada RA, Fujiwara K, et al. Coordination of cellular localization-dependent effects of SUMOylation in regulating cardiovascular and neurological diseases. Adv Exp Med Biol [Internet]. 2017 [cited 2023 Jan 16];963:337–58. Available from: https://www.ncbi.nlm.nih.gov/pmc/articles/PMC5716632/. doi: 10.1007/978-3-319-50044-7_20 28197922 PMC5716632

[67] Rosonina E, Akhter A, Dou Y, Babu J, Sri Theivakadadcham VS. Regulation of transcription factors by sumoylation. Transcription [Internet]. 2017 [cited 2023 Jan 16];8:220–31. Available from: https://www.ncbi.nlm.nih.gov/pmc/articles/PMC5574528/. doi: 10.1080/21541264.2017.1311829 28379052 PMC5574528

[68] Yang Y, He Y, Wang X, liang Z, He G, Zhang P, et al. Protein SUMOylation modification and its associations with disease. Open Biology [Internet]. 2017 [cited 2023 Jan 16];7:170167. Available from: https://royalsocietypublishing.org/doi/10.1098/rsob.170167. 29021212 10.1098/rsob.170167 PMC5666083

[69] Hofmann WA, Arduini A, Nicol SM, Camacho CJ, Lessard JL, Fuller-Pace FV, et al. SUMOylation of nuclear actin. J Cell Biol [Internet]. 2009 [cited 2023 Jan 16];186:193–200. Available from: https://www.ncbi.nlm.nih.gov/pmc/articles/PMC2717643/. doi: 10.1083/jcb.200905016 19635839 PMC2717643

[70] Feng W, Liu R, Xie X, Diao L, Gao N, Cheng J, et al. SUMOylation of α-tubulin is a novel modification regulating microtubule dynamics. Journal of Molecular Cell Biology [Internet]. 2021 [cited 2023 Jan 16];13:91–103. Available from: 10.1093/jmcb/mjaa076. 33394042 PMC8104938

[71] van Niekerk EA, Willis DE, Chang JH, Reumann K, Heise T, Twiss JL. Sumoylation in axons triggers retrograde transport of the RNA-binding protein La. Proceedings of the National Academy of Sciences [Internet]. 2007 [cited 2023 Jan 16];104:12913–8. Available from: https://www.pnas.org/doi/10.1073/pnas.0611562104. 17646655 10.1073/pnas.0611562104 PMC1937566

[72] Barthel M, Hapfelmeier S, Quintanilla-Martínez L, Kremer M, Rohde M, Hogardt M, et al. Pretreatment of mice with streptomycin provides a Salmonella enterica serovar Typhimurium colitis model that allows analysis of both pathogen and host. Infect Immun. 2003;71:2839–58. doi: 10.1128/IAI.71.5.2839-2858.2003 12704158 PMC153285

[73] Auerbuch V, Lenz LL, Portnoy DA. Development of a competitive index assay to evaluate the virulence of Listeria monocytogenes actA mutants during primary and secondary infection of mice. Infect Immun. 2001;69:5953–7. doi: 10.1128/IAI.69.9.5953-5957.2001 11500481 PMC98721

