# Supplementary figures and images for: SifA SUMOylation governs Salmonella Typhimurium intracellular survival via modulation of lysosomal function
Source: PLoS Pathog. 2023 Sep 29;19(9):e1011686. doi: 10.1371/journal.ppat.1011686 (PMC10566704; doi:10.1371/journal.ppat.1011686)

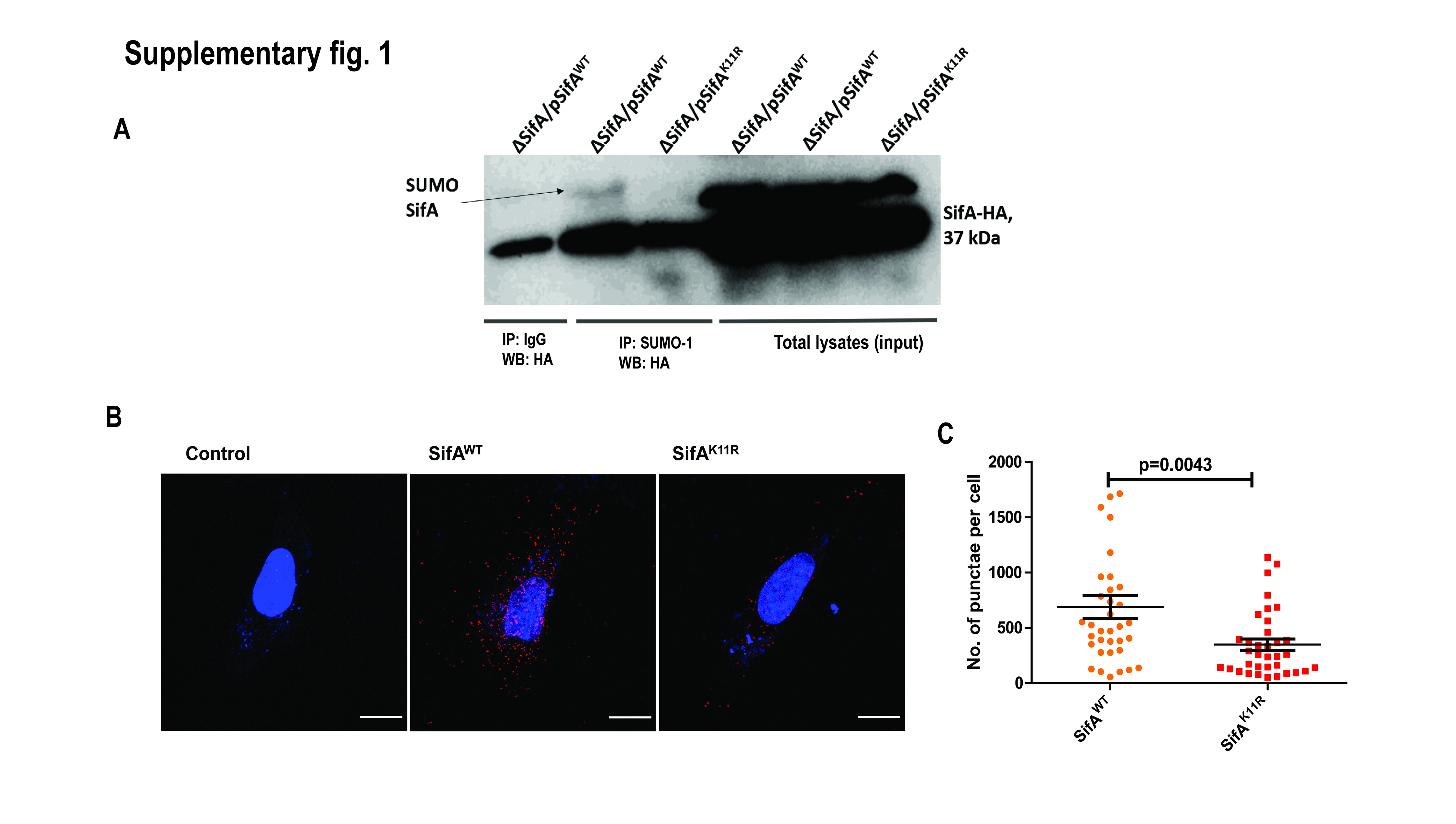

Supplement: S1 Fig — (A) Co-immunoprecipitation of SUMOylated SifA from HCT-8 cells during infection by complemented strains pSifAWT and pSifAK11R expressing SifA-HA. IP was carried from infected protein lysates 7 hours post infection by SUMO-1 antibody followed by immunoblotting by anti-HA antibody. A faint band corresponding to SUMOylated SifA shown was obtained only in pSifAWT lane. This is a representative blot of single experiment. (B) Images displaying discrete PLA punctae representing co-localization of SifA and SUMO-1 in indicated samples (scale 6 micron). (C) Quantification of PLA punctae carried out using particle analysis tool from Image J software. (TIF) [file ppat.1011686.s001.tif]

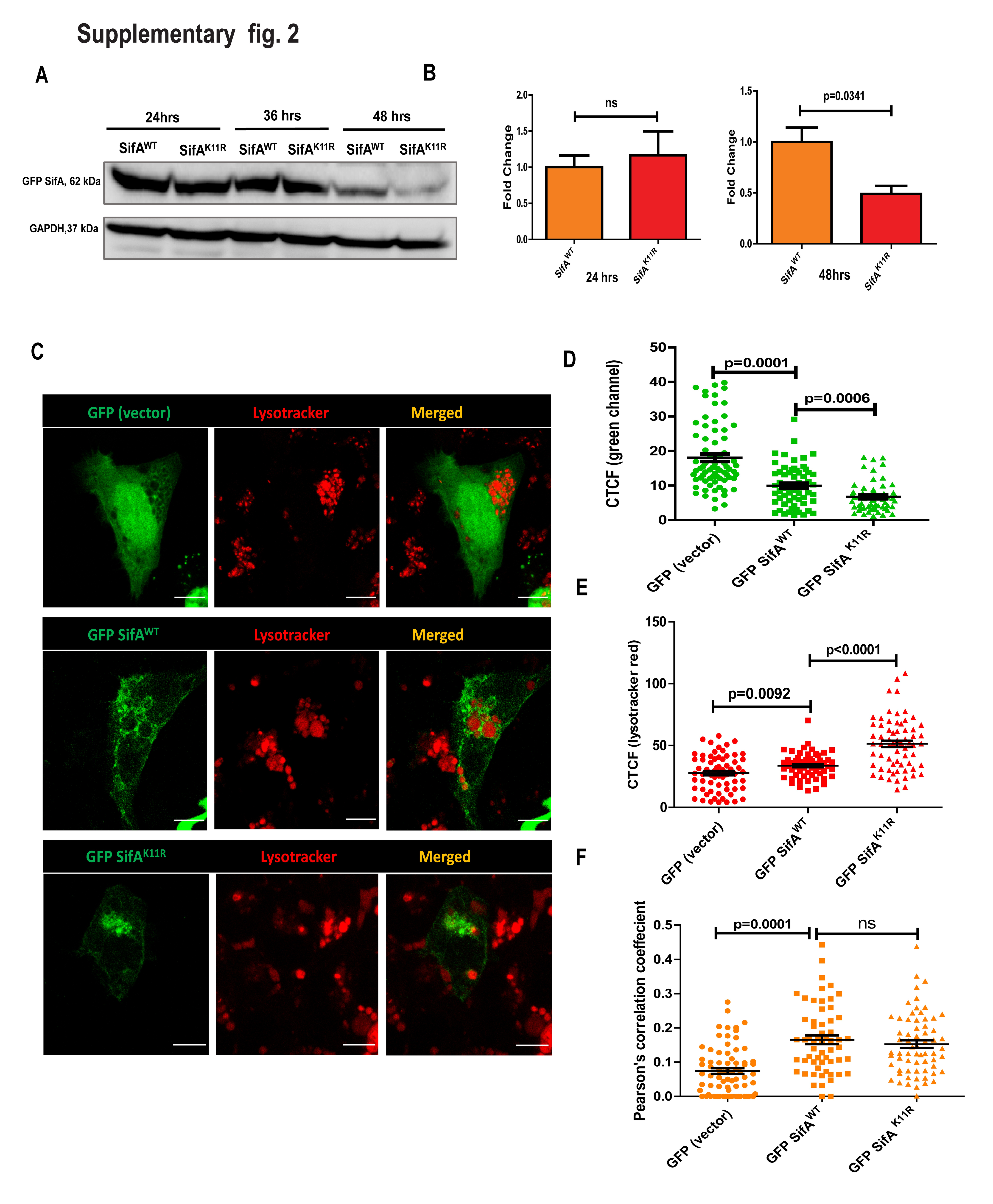

Supplement: S2 Fig — (A) Timeline of expression levels of ectopically expressed SifAWT and SifAK11R proteins from HCT-8 cell lysates. (B) Lysates prepared after 48 hours of transfection, showed a significant change in expression. (C) Live cell snapshots of transfected HCT-8 cells indicating localization of GFP SifAWT, GFP SifAK11R, and empty vector with lysosomes labeled by lysotracker red (scale 8 microns). Corrected image sum intensity quantified for (D) GFP and (E) lysotracker red fluorescence. (F) Co-localization analysis between both channels (GFP and lysotracker) for all three transfected conditions are also performed. The blots shown here are representative of at least three biological replicates. (TIF) [file ppat.1011686.s002.tif]

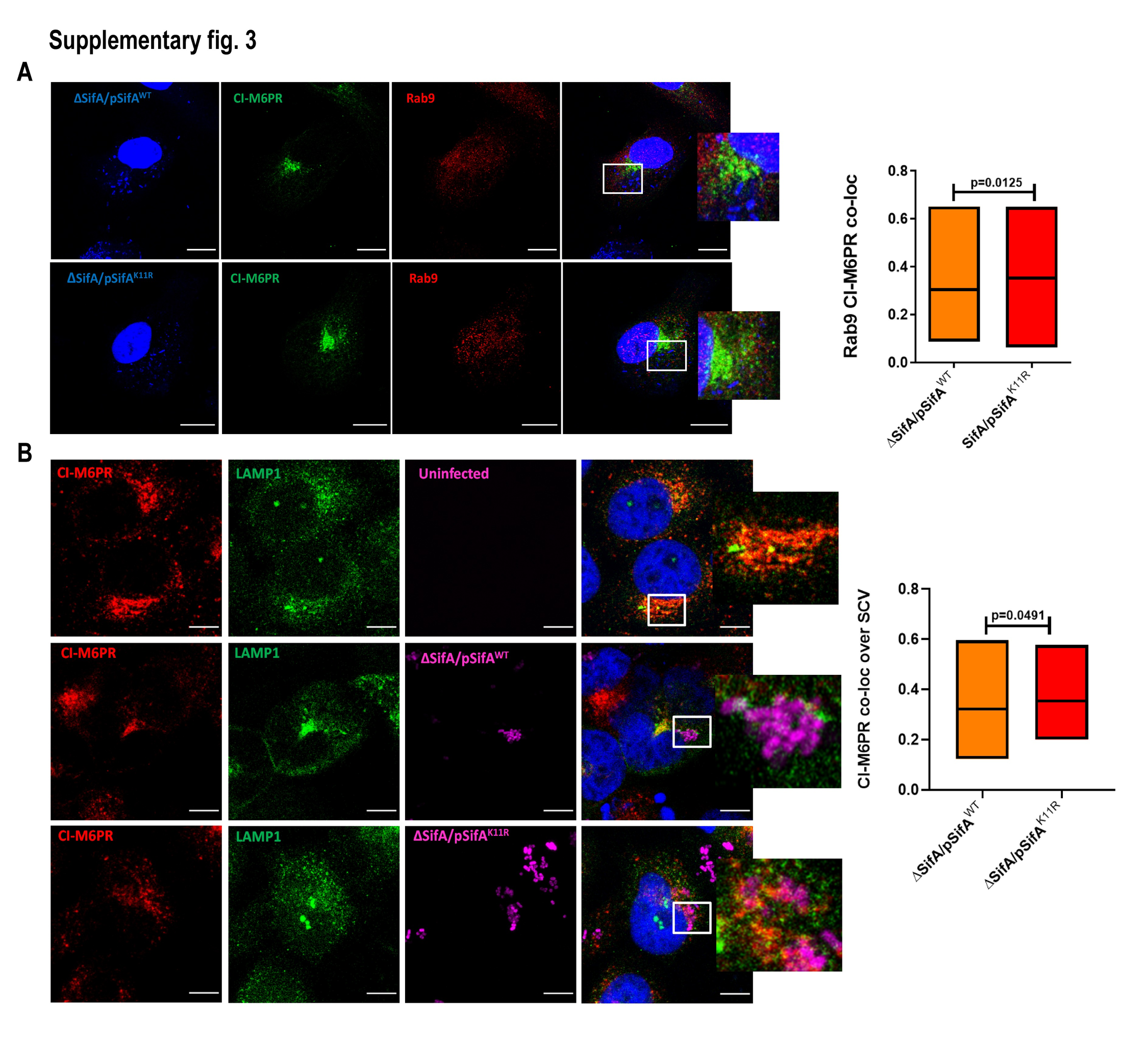

Supplement: S3 Fig — (A) Images co-stained for Rab9 and CI-M6PR for co-localization analysis in HeLa cells under pSifAWT (scale 9 micron) and pSifAK11R (scale 11 microns) infection conditions. (B) Distribution and quantization of CI-M6PR over SCVs from similar infection conditions (scale 10 microns). SCV marker LAMP1 was used to indicate SCVs. Salmonella used here expresses mCherry plasmid for visualization. (TIF) [file ppat.1011686.s003.tif]

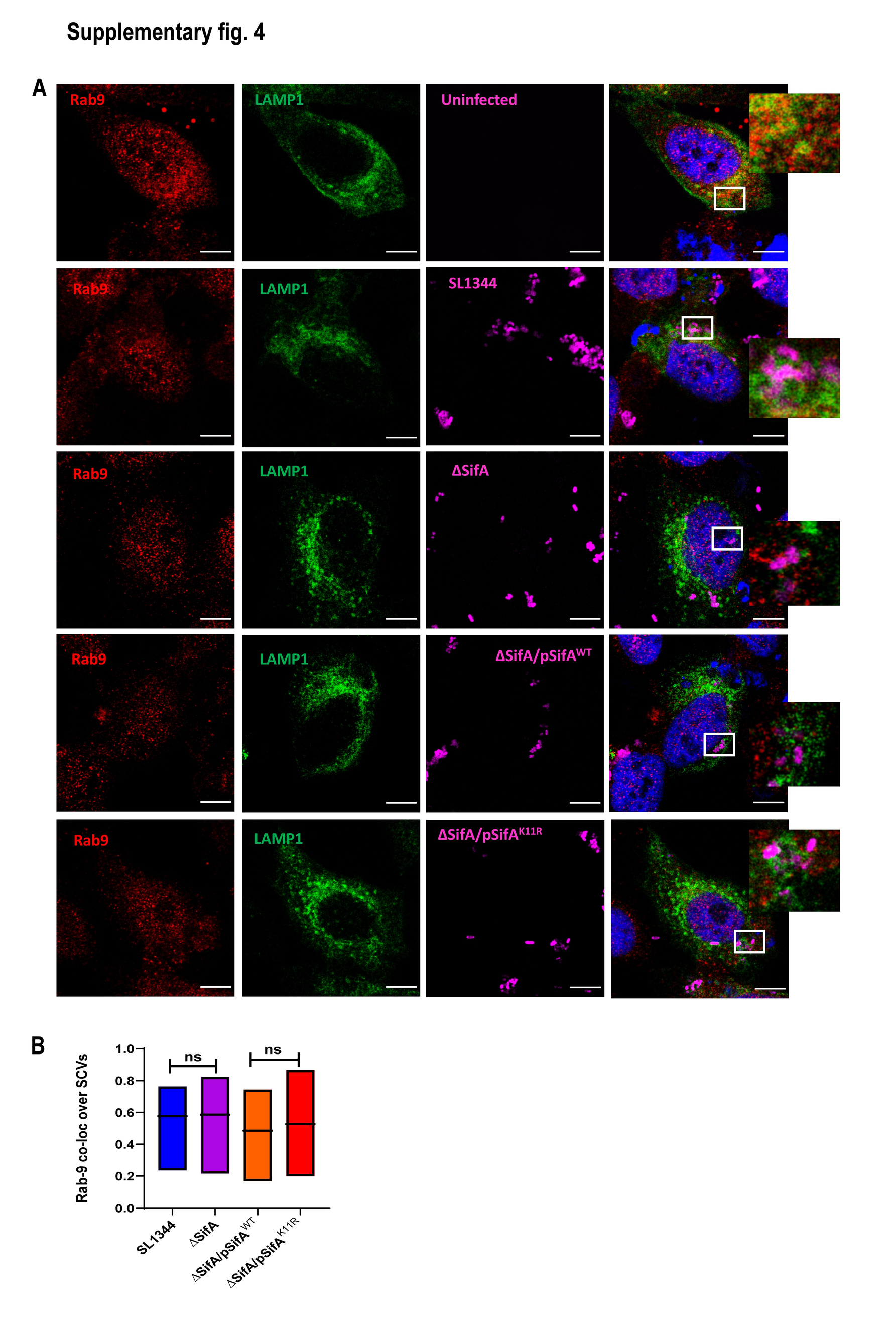

Supplement: S4 Fig — (A) Images co-stained for Rab9 and LAMP1 to analyze Rab9 co-localization over SCVs from infected HeLa cells by depicted strains expressing mCherry (scale 10 microns). (B) Quantification of co-localization of Rab9 over SCVs from acquired images. (TIF) [file ppat.1011686.s004.tif]

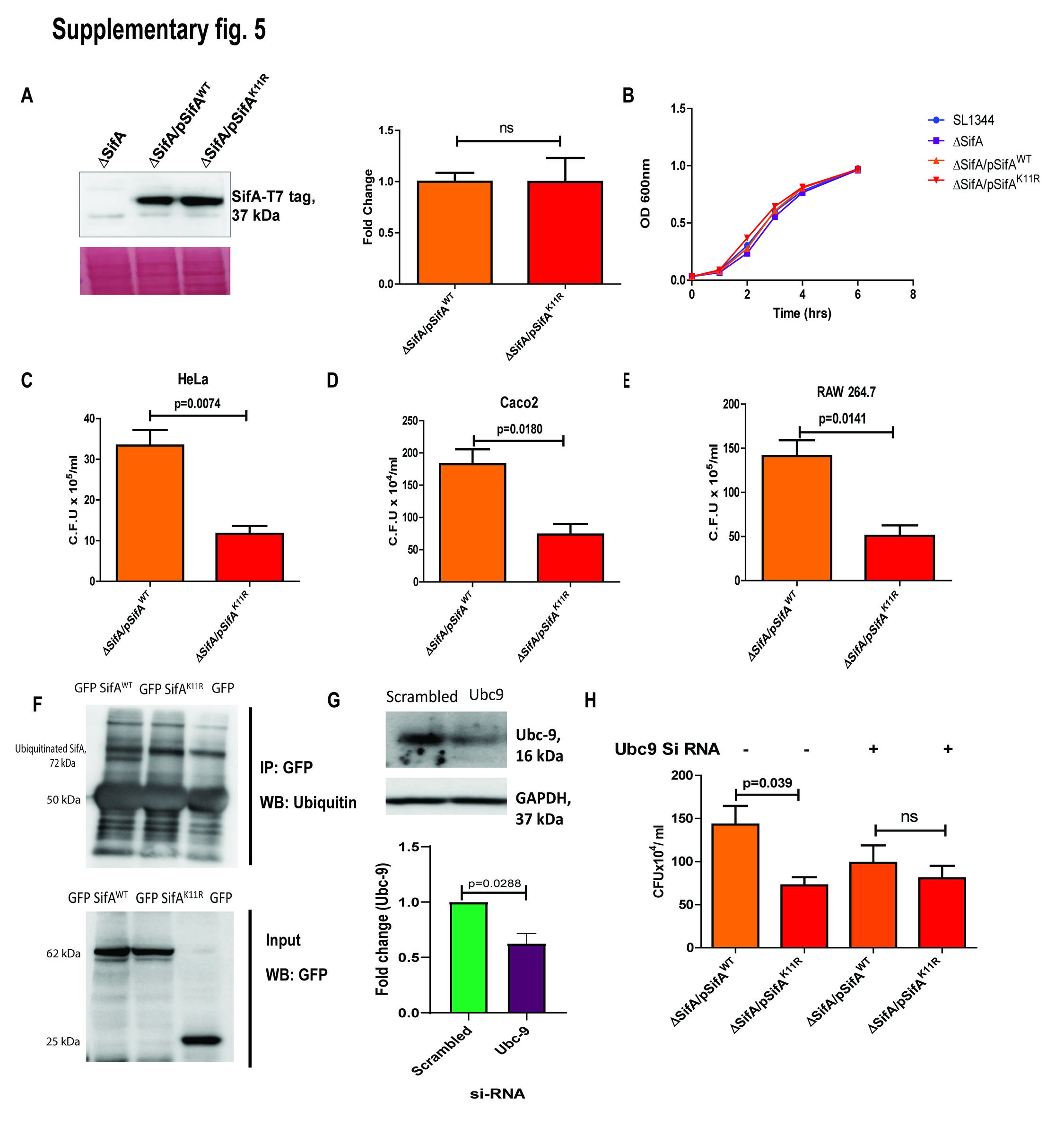

Supplement: S5 Fig — (A) The expression levels of SifAWT and SifA K11R proteins from ΔSifA complemented with SifAWT (SifAWT) and SifAK11R (SifAK11R) respectively. (B) The growth curve indicating division rate of strains SL1344, ΔSifA, SifAWT and SifAK11R. Comparison of CFU results obtained from SifAWT and SifAKIIR infected (C) HeLa (D) Caco2 cells and (E) RAW 264.7cells post 16 hours of infection. (F) Interaction of transfected GFP SifA with ubiquitin from HCT-8 cells. SifA is shown to be ubiquitinated at lysine 11 by co-IP assay using GFP antibody, followed by immunoblotting using both ubiquitin and GFP antibodies. (G) Expression levels of Ubc-9 in presence of scrambled and Ubc-9 siRNA. (H) Replication rates of SifAWT and SifAK11R strains under Ubc-9 knockdown conditions. The blots shown here are representative of at least three biological replicates. (TIF) [file ppat.1011686.s005.tif]
